# Distinct patterns and processes of abundant and rare eukaryotic plankton communities following a reservoir cyanobacterial bloom

**DOI:** 10.1038/s41396-018-0159-0

**Published:** 2018-06-13

**Authors:** Yuanyuan Xue, Huihuang Chen, Jun R. Yang, Min Liu, Bangqin Huang, Jun Yang

**Affiliations:** 1 Aquatic EcoHealth Group, Key Laboratory of Urban Environment and Health, Institute of Urban Environment, Chinese Academy of Sciences, Xiamen, 361021 China; 2 University of Chinese Academy of Sciences, Beijing, 100049 China; 3 College of Environment and Ecology, Xiamen University, Xiamen, 361102 China

## Abstract

Plankton communities normally consist of few abundant and many rare species, yet little is known about the ecological role of rare planktonic eukaryotes. Here we used a 18S ribosomal DNA sequencing approach to investigate the dynamics of rare planktonic eukaryotes, and to explore the co-occurrence patterns of abundant and rare eukaryotic plankton in a subtropical reservoir following a cyanobacterial bloom event. Our results showed that the bloom event significantly altered the eukaryotic plankton community composition and rare plankton diversity without affecting the diversity of abundant plankton. The similarities of both abundant and rare eukaryotic plankton subcommunities significantly declined with the increase in time-lag, but stronger temporal turnover was observed in rare taxa. Further, species turnover of both subcommunities explained a higher percentage of the community variation than species richness. Both deterministic and stochastic processes significantly influenced eukaryotic plankton community assembly, and the stochastic pattern (e.g., ecological drift) was particularly pronounced for rare taxa. Co-occurrence network analysis revealed that keystone taxa mainly belonged to rare species, which may play fundamental roles in network persistence. Importantly, covariations between rare and non-rare taxa were predominantly positive, implying multispecies cooperation might contribute to the stability and resilience of the microbial community. Overall, these findings expand current understanding of the ecological mechanisms and microbial interactions underlying plankton dynamics in changing aquatic ecosystems.

## Introduction

Disturbance can have profound and multiple effects on ecosystems, greatly altering natural community structure [1]. In aquatic systems, cyanobacterial blooms are a common biological disturbance affecting plankton communities [2]. Eukaryotic plankton play an important role in the trophic web structure [3], and cyanobacterial blooms may change food webs by inhibiting the growth of other phytoplankton, further influencing resource use efficiency in phytoplankton and zooplankton communities [4, 5]. As important components of food webs, eukaryotic plankton carry out a range of ecological roles, such as primary producers, bacterivores, planktivores, parasites, and saprotrophs [6, 7]. Thus their responses to environmental fluctuations may directly influence aquatic ecosystem functioning [8]. However, there are significant knowledge gaps about how environmental conditions shape whole eukaryotic plankton communities (including Protists and Fungi) [9]. Reservoirs are one of suitable freshwater ecosystems in which to study the succession of eukaryotic plankton communities, owing to their highly dynamic environment dominated by diverse microorganisms [10].

Eukaryotic plankton are extremely diverse with relatively few abundant species co-existing with a large number of rare species, the latter often referred to as the “rare biosphere” [11, 12]. Owing to methodical limitations, previous analyses of eukaryotic plankton communities have mainly focused on relatively abundant taxa through microscope observation [13]. These abundant taxa contribute to the fluxes of organic matter and biomass production [14], thereby studying their community composition is important for understanding ecological function. Rapid advances in molecular approaches have substantially boosted our knowledge of rare microbial diversity. In particular, high-throughput sequencing technologies have been successfully applied to compare the composition and dynamics of abundant and rare eukaryotic communities in various aquatic ecosystems, including coastal waters and intertidal sediments [15], epipelagic waters [16], and reservoirs [17].

Recent studies have increasingly emphasized the ecological importance of the rare biosphere, because rare taxa can include more metabolically active microorganisms than abundant taxa (as measured by RNA to DNA ratios), and they may be keystone species in regulating the functioning of aquatic environments [18, 19]. The rare microbes have been shown to fulfill essential functions associated with nutrient cycling, and may enhance functionality of the abundant microbes (as reviewed in Jousset et al. [20]). Additionally, as part of the microbial “seed bank”, rare taxa can potentially drive ecosystem responses to environmental changes and become dominant under favorable conditions [21], therefore providing a mechanism for community persistence and stability [22]. In aquatic ecosystems, the response of plankton communities to environmental change is mediated by their properties, such as physiological tolerance, dispersal capacity, and taxonomic, and functional diversity [8, 23]. Abundant and rare community assemblages are likely subject to different controlling factors. For example, previous marine studies showed that in surface waters, abundant taxa were primarily influenced by dispersal limitations, while rare taxa were mainly controlled by environmental filtering [16].

Cyanobacterial blooms can have a major impact on the microbial community through both direct (e.g., species interactions) and indirect (e.g., bloom-induced changes in water properties) effects [2, 24]. While there has been considerable research conducted on the interactions between the abundant planktonic eukaryotes and cyanobacteria [25, 26], few studies have investigated how cyanobacterial blooms affect rare planktonic eukaryotes in the water column. A complex network of interrelationships can reveal the intrinsic mechanisms of microbial interactions in response to environmental disturbance [27]. When considering the whole eukaryotic community, the complex co-occurrence networks between interacting microorganisms (e.g., autotrophs, heterotrophs, and parasites; abundant and rare taxa) and the topological features of these networks can be explored. Such networks have been constructed to unveil bacterial interactions in a range of systems, such as river [28], oil-contaminated soil [29], and marine [30] ecosystems. Until now, however, the co-occurrence patterns of rare and abundant eukaryotes, particularly in reservoir and lake ecosystems, have not been investigated.

Here, for the first time, we investigated the temporal patterns of eukaryotic plankton communities using 18S rRNA gene-based high-throughput sequencing and explored the associations between abundant and rare planktonic eukaryotes based on network analysis in a subtropical reservoir. This reservoir system was experiencing a cyanobacterial bloom at the beginning of the sampling period, and we tracked the biotic and abiotic changes through the three months following the bloom event. Our main objectives are to (i) compare the diversity and composition of abundant and rare eukaryotic plankton communities, and their relative contributions to community shift over time; (ii) uncover the co-occurrence patterns of abundant and rare planktonic eukaryotes; (iii) identify the controlling mechanisms and factors that influence the dynamics of the eukaryotic plankton community. Taken together, this research will enhance our understanding of the dynamics of eukaryotic plankton communities in variable environments, with special emphasis on the rare biosphere.

## Materials and methods

### Sampling and environmental information

The study site (Xidong Reservoir, 24°49′ N, 118°10′ E), and the sampling design have been previously described in Xue et al. [24]. Briefly, water samples (*n* = 18) were collected twice a month from October to December 2014 across three discrete depths: surface (0.5 m), middle (thermocline or oxycline at 12, 14, 17, 17, 20, and 18 m, for the six sampling dates) and bottom (25 m) water layers. The cyanobacterial bloom was observed in October, and the reservoir recovered in November based on chlorophyll *a* concentration and water transparency (Fig. S1). Microscopic inspection showed that this bloom was almost exclusively (>80% of phytoplankton biomass) composed of *Microcystis aeruginosa* (Fig. S2). A temporal frame work divided into three successional periods is used here: the bloom period in October, post-bloom period 1 in November, and post-bloom period 2 in December. Water samples were divided into two subsamples: one for eukaryotic plankton community analyses and the other for water chemistry. For the eukaryotic plankton, 500 mL of water was filtered through 0.22 μm polycarbonate filters (47 mm diameter, Millipore, Billerica, MA, USA). Filters were stored at –80 °C until further processing. In total, 17 measured environmental variables in the water were taken directly from our previous study [24]. Transparency was determined with a 30 cm Secchi disk. Chlorophyll *a* was measured with a PHYTO-PAM Phytoplankton Analyzer (Heinz Walz GmbH, Eichenring, Germany).

### DNA extraction, PCR, and Illumina sequencing

Extraction and purification of plankton DNA from filters were carried out using the FastDNA spin kit for soil (MP Biomedicals, Santa Ana, CA, USA) following the manufacturer’s instructions. The V9 region of the eukaryotic small-subunit 18S rRNA gene was amplified using the primer set 1380F (5′-CCCTGCCHTTTGTACACAC-3′) and 1510R (5′-CCTTCYGCAGGTTCACCTAC-3′) [31]. Each plankton DNA sample was PCR-amplified in triplicate. The 30 μL PCR mixture contained 15 μL of Phusion High-Fidelity PCR Master Mix (New England Biolabs, Beverly, MA, USA), 0.2 μM of each primer, and 10 ng of sample DNA. The PCR reaction conditions were 98 °C for 1 min, followed by 30 cycles of 10 s at 98 °C, 50 °C for 30 s, 72 °C for 30 s, and a final extension at 72 °C for 5 min. The triplicate PCR products for each sample were pooled in equal quantity for purification and sequenced on the Illumina HiSeq2500 platform (Illumina, Inc., San Diego, CA, USA) using a paired-end (2×250 bp) approach [17].

### Bioinformatics

Paired-end reads from the raw DNA fragments were assembled with FLASH and the mean contig read length was 137 ± 0.6 bp [32]. For the 18 water samples, we obtained 2,532,735 raw reads, ranging from 127,914 to 155,100 with a mean of 140,707 reads per sample. Sequence processing was performed using the quantitative insights into microbial ecology (QIIME v.1.8.0) pipeline with the following settings: reads with an average Phred score <25 were discarded; maximum number of consecutive low-quality base = 3; minimum of continuous high-quality base = 75% of total read length; maximum number of ambiguous bases = 0, last quality score = 3 [33]. Chimeric sequences were identified by UCHIME and removed before downstream analysis [34]. The UPARSE pipeline was used to pick operational taxonomic units (OTUs) at 97% similarity level [35]. Subsequently, a representative sequence from each OTU was aligned against the SILVA 123 reference alignment using the RDP classifier [36]. To minimize inclusion of sequencing errors, singletons (OTUs with only one sequence) were eliminated in the whole data set, and then we used a randomly selected subset of 123,090 sequences from each sample to normalize sequencing effort across samples. The final total data set retained 2305 OTUs and 2,215,620 reads at 97% similarity level.

### Definition of abundant and rare taxa

The definition of abundant and rare taxa depends on the cutoff level of relative abundance, setting 0.1 or 0.01% as rare OTUs and 1% as abundant OTUs [11, 15, 17]. These classifications can neglect the intermediate taxa (i.e., relative abundance between 0.1 or 0.01 and 1%) and the oscillating taxa (i.e., rare and abundant under different conditions). In this study, we classified all OTUs into six categories following recent studies [12, 37]: always abundant taxa (AAT) with a relative abundance ≥1% in all samples; conditionally abundant taxa (CAT) with a relative abundance ≥0.01% in all samples and ≥1% in some samples; always rare taxa (ART) with a relative abundance <0.01% in all samples; conditionally rare taxa (CRT) with a relative abundance <0.01% in some samples but never ≥1% in any sample; moderate taxa with a relative abundance between 0.01% and 1% in all samples (MT); conditionally rare and abundant taxa (CRAT) with a relative abundance ranging from rare (<0.01%) to abundant (≥1%). Then, for the comparative study of abundant and rare taxa, the abundant taxa (AT) consisted of always abundant and conditionally abundant taxa, and the rare taxa (RT) composed of always rare and conditionally rare taxa. Detailed and general descriptions of abundant and rare data sets are presented in Supplementary Tables S1 and S2.

### Ecological inference

We tentatively performed a basic functional annotation to discriminate OTUs corresponding to pigmented and non-pigmented taxa. Ecological inference was based on the literature describing the presence or absence of chloroplasts in microbial eukaryotic groups [3, 38, 39]. Pigmented groups comprised both obligate phototrophs (e.g., Chlorophyta and Diatomea) and mixotrophs (e.g., Chrysophyceae, Cryptophyceae, and Dinophyceae). OTUs distributed within groups of free-living heterotrophs (e.g., Ciliophora, Cercozoa, and osmotrophic Fungi) and parasites (Apicomplexa, Chytridiomycota, Perkinsidae, Peronosporomycetes, and Pirsonia) were reliably assigned to non-pigmented taxa. When OTUs could not be precisely identified (i.e., to at least the genus level), they were assigned to “unknown” ecological roles (e.g., unidentified Alveolata).

### Statistical analyses

#### Alpha-diversity analysis

Alpha-diversity indices were computed using the *diversity* function in the “vegan” R package. Spatiotemporal effects on alpha-diversity were examined with two-way analysis of variance (two-way ANOVA) using SPSS Statistics for Windows v.22.0 (IBM Corp., Armonk, NY, USA). To estimate sampling effort, we fitted our data to a truncated Preston log-normal distribution using the *prestonfit* and *prestondistr* functions of the “vegan” R package [40].

#### Beta-diversity analysis

Beta-diversity was measured using Bray–Curtis dissimilarity [41]. The beta-diversity values of different subcommunities were partitioned into two components, the balanced variation (richness) and abundance gradient (turnover), using the *bray.part* function of the “betapart” R package [29]. To reveal temporal patterns in eukaryotic plankton communities, a time-lag analysis was used to quantify the Bray–Curtis dissimilarity between each pair of samples, and the time difference (lag) was then plotted against the dissimilarity [42]. Eukaryotic plankton community composition was visualized using non-metric multidimensional scaling (NMDS) based on Bray–Curtis dissimilarities. Analysis of similarity (ANOSIM) was used to investigate differences in eukaryotic plankton communities between groups. The contribution of each species (i.e., OTU) to community dissimilarity over time was calculated using similarity percentage (SIMPER) analysis. The NMDS, ANOSIM, and SIMPER analyses were performed using PRIMER v.7.0 and PAST v.2.12. Finally, to further explain the patterns of beta-diversity, we calculated Levin’s niche breadth index separately for the abundant and rare taxa [43].

#### Indicator OTUs associated with environmental changes

We determined which OTUs could explain the time effect in diversity by identifying indicator species in the “indicspecies” R package [44]. This analysis calculates an indicator value that measures the association between OTUs and groups of samples and then identifies the group corresponding to the highest association value. For this study, we compared samples across three periods (cyanobacterial bloom period, post-bloom period 1, and post-bloom period 2) to determine temporal indicators based on an indicator value >0.7 and *P*-value < 0.05 assessed after 999 permutation tests.

#### Network analysis

To reduce the complexity of the data sets, OTUs present in more than six samples with more than 20 sequences were retained for the construction of networks. A total of 1058 OTUs were used for the network analyses. Subsequently, all possible pairwise Spearman’s rank correlations (*r*) between those OTUs were calculated within the “picante” R package. Only robust (*r* > 0.8 or *r* < −0.8) and statistically significant (*P*-value < 0.01) correlations were incorporated into network analyses [28]. Network visualization and modular analysis were made with Gephi version 0.8.2. Node-level topological properties (i.e., degree, betweenness, closeness, and eigenvector) were further calculated in the “igraph” R package. Statistical differences in measured node-level attributes across different taxa were determined using nonparametric Mann–Whitney *U* test. Nodes with high degree (>100) and low betweenness centrality values (<5000) are recognized as keystone species in co-occurrence networks [45]. Hubs (i.e., highly linked species within their own module) and connectors linking different modules in co-occurrence network were identified based on their connectivity as described previously [46]. Meanwhile, 1000 Erdös–Réyni random networks, which had the identical number of nodes and edges as the real networks, were generated in the “igraph” R package, with each edge having the same probability of being assigned to any node [47]. Topology characteristics of both real and random networks were calculated and compared, including modularity, clustering coefficient, and average path length.

#### Relationships between community composition and environment variables

We implemented the Mantel test using the “vegan” package in R to select significant environmental factors correlated with the variations of eukaryotic plankton communities. Prior to the analysis, the normality of the physicochemical variables was checked using the Shapiro–Wilk test, and log(*x*+1) transformed, with the exception of pH, to improve normality and homoscedasticity. Additionally, Spearman correlations were calculated to discern the relationship between the OTUs of each major module and environmental variables in the “picante” R package. Only robust (Spearman’s *r* > 0.8 or *r* < −0.8) and statistically significant (*P*-value < 0.01) correlations were considered in this study.

#### Neutral community model

A neutral community model was used to determine the potential contribution of neutral processes to eukaryotic plankton community assembly by predicting the relationship between OTUs occurrence frequency and their relative abundance [48]. This model predicts that rare taxa will be lost with time due to ecological drift, while abundant taxa are more likely to be dispersed by chance and thus present in more samples. In this model, *R*^2^ and Nm values indicate the fit to the neutral model and metacommunity size times immigration, respectively.

### Accession numbers

All raw sequences data from this study have been submitted to the NCBI Sequence Read Archive (SRA) database under the BioProject number PRJNA348137 and the accession number SRP091963.

## Results

### General patterns of species richness and alpha-diversity

Overall, the sequencing of 18S rRNA genes yielded 2,215,620 high-quality sequences and 2305 OTUs at 97% similarity level. The total number of OTUs (2305) in all samples was roughly equivalent to the richness estimated by Chao 1 (2372 ± 15) and ACE (2371 ± 24) (Table S2). The rarefaction curves almost approached saturation for the total and three separate periods (Fig. S3a). Further, we fitted our community data to the truncated Preston log-normal model (Fig. S3b), and estimated that our sampling found 87–88% of the OTUs in the studied reservoir. These results indicated that the majority of the eukaryotic plankton diversity had been recovered by the deep sequencing.

In the whole data set, 44 OTUs (1.91%) representing 73.04% of all sequences were abundant and persistent across all periods, whereas 2167 OTUs (94.01%) contributing 13.84% of all sequences were affiliated to rare taxa (Table S2). The non-pigmented taxa (mainly Ciliophora, Fungi, and Cercozoa) dominated the OTU richness, together representing 40.5% of the total OTUs (Table S3). Pigmented groups, dominated by Chlorophyta (117 OTUs), Chrysophyceae (99 OTUs), and Cryptophyceae (94 OTUs), accounted for only 20.7% of all OTUs.

**Fig. 1:**
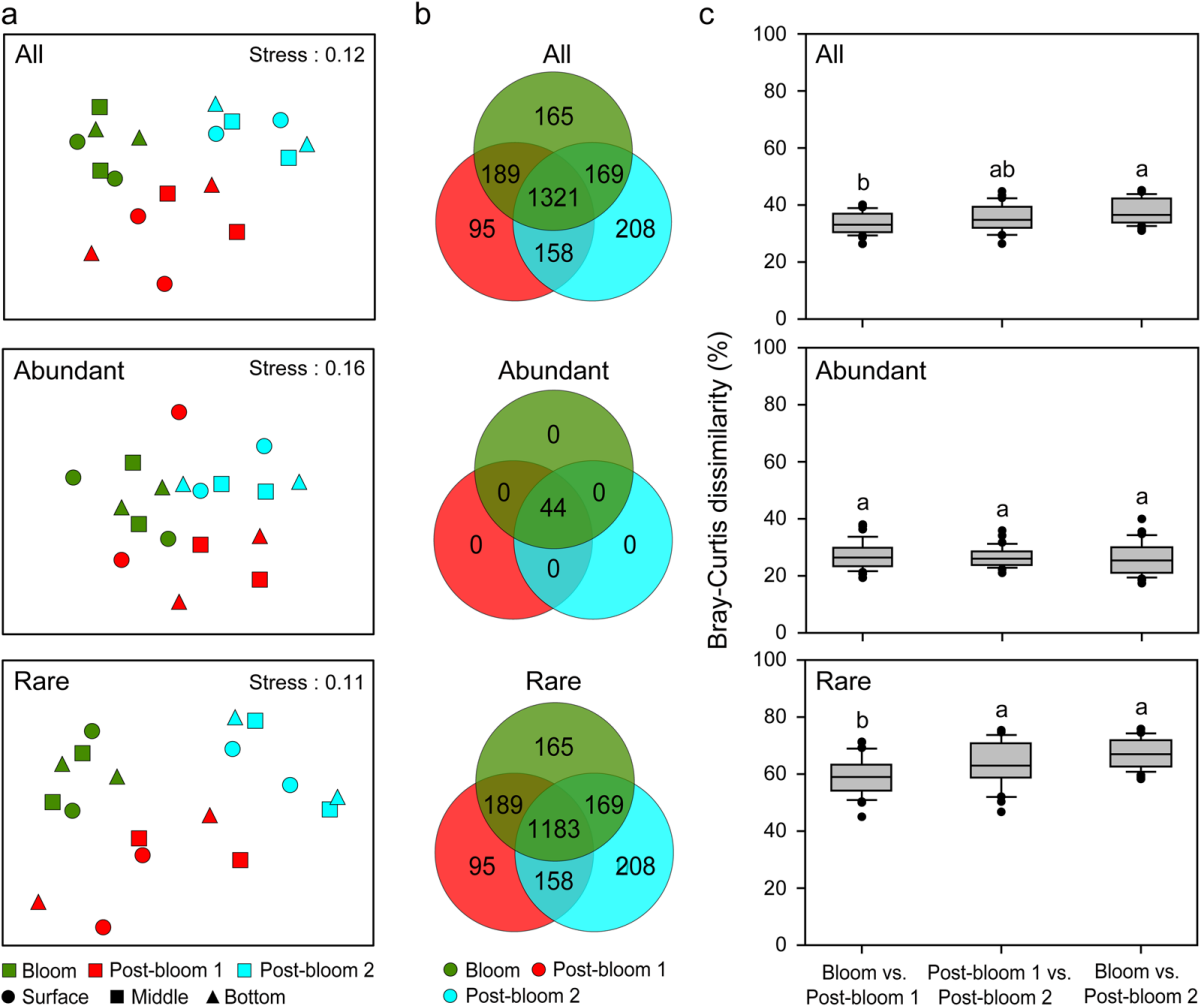
Community structuring of eukaryotic plankton across bloom event. **a** Non-metric multidimensional scaling (NMDS) ordination of eukaryotic plankton communities based on Bray–Curtis distances. **b** Venn diagram showing the numbers of unique and shared OTUs between three different periods. **c** The pairwise Bray–Curtis dissimilarity of eukaryotic plankton communities between different periods. The top and bottom boundaries of each box indicate the 75th and 25th quartile values, respectively, and lines within each box represent the median values (*n* = 36). Different letters above bars indicate a significant difference at the *P* < 0.05 level according to nonparametric Mann-Whitney *U* test. All, whole eukaryotic plankton communities; Abundant, abundant plankton subcommunities; Rare, rare plankton subcommunities

Alpha-diversity of all and abundant taxa showed a similar pattern, and did not differ over depth and time (Table 1). However, rare taxa exhibited a significant temporal pattern with an increase in Shannon–Wiener diversity, Simpson diversity, and Pielou’s evenness from bloom to post-bloom 1, followed by a loss of these indices during post-bloom 2 (Table 1 and Fig. S4). Venn diagram analysis indicated that most of the OTUs were shared among three periods, and all of the period-specific OTUs belonged to rare taxa (Fig. 1b). Additionally, we found high variability in the number of strict indicator OTUs over time (ranging from 41 during bloom period, 3 during post-bloom period 1 to 87 during post-bloom period 2). The taxonomic compositions of strict indicators were significantly different among three different periods (Table S4).

**Table 1:** Two-way ANOVA showing the effects of time and depth on the alpha-diversity of eukaryotic plankton

	All		Abundant		Rare	
	*F*	*P*	*F*	*P*	*F*	*P*
Time						
Richness	1.789	0.222			1.792	0.221
ACE	1.674	0.241			1.678	0.240
Chao 1	1.646	0.246			1.658	0.244
Shannon–Wiener	1.264	0.328	1.025	0.397	11.561	**0.003**
Simpson	1.373	0.302	1.382	0.300	9.422	**0.006**
Pielou’s evenness	1.333	0.311	1.025	0.397	20.786	**0.000**
Depth						
Richness	1.356	0.306			1.353	0.306
ACE	1.166	0.354			1.170	0.354
Chao 1	1.378	0.301			1.384	0.299
Shannon–Wiener	1.419	0.291	1.366	0.303	0.571	0.584
Simpson	1.806	0.219	1.492	0.276	0.142	0.870
Pielou’s evenness	1.437	0.287	1.366	0.303	0.016	0.984
Time × Depth						
Richness	1.814	0.210			1.802	0.213
ACE	1.470	0.289			1.452	0.294
Chao 1	1.511	0.278			1.508	0.279
Shannon–Wiener	1.207	0.372	0.857	0.525	0.357	0.833
Simpson	0.865	0.520	0.686	0.619	0.567	0.693
Pielou’s evenness	1.125	0.403	0.857	0.525	0.188	0.939

Note that abundant OTUs were persistent across all samples, and the *F* and *P* values of richness, ACE, and Chao 1 indices cannot be calculated

Time indicates three successional periods, including bloom, post-bloom 1, and post-bloom 2 periods

Depth denotes surface, middle, and bottom water layers

All, whole eukaryotic plankton; Abundant, abundant eukaryotic plankton; Rare, rare eukaryotic plankton

Bold font means the significance at *P* < 0.05 level

### Temporal dynamics in microbial community composition

**Fig. 2:**
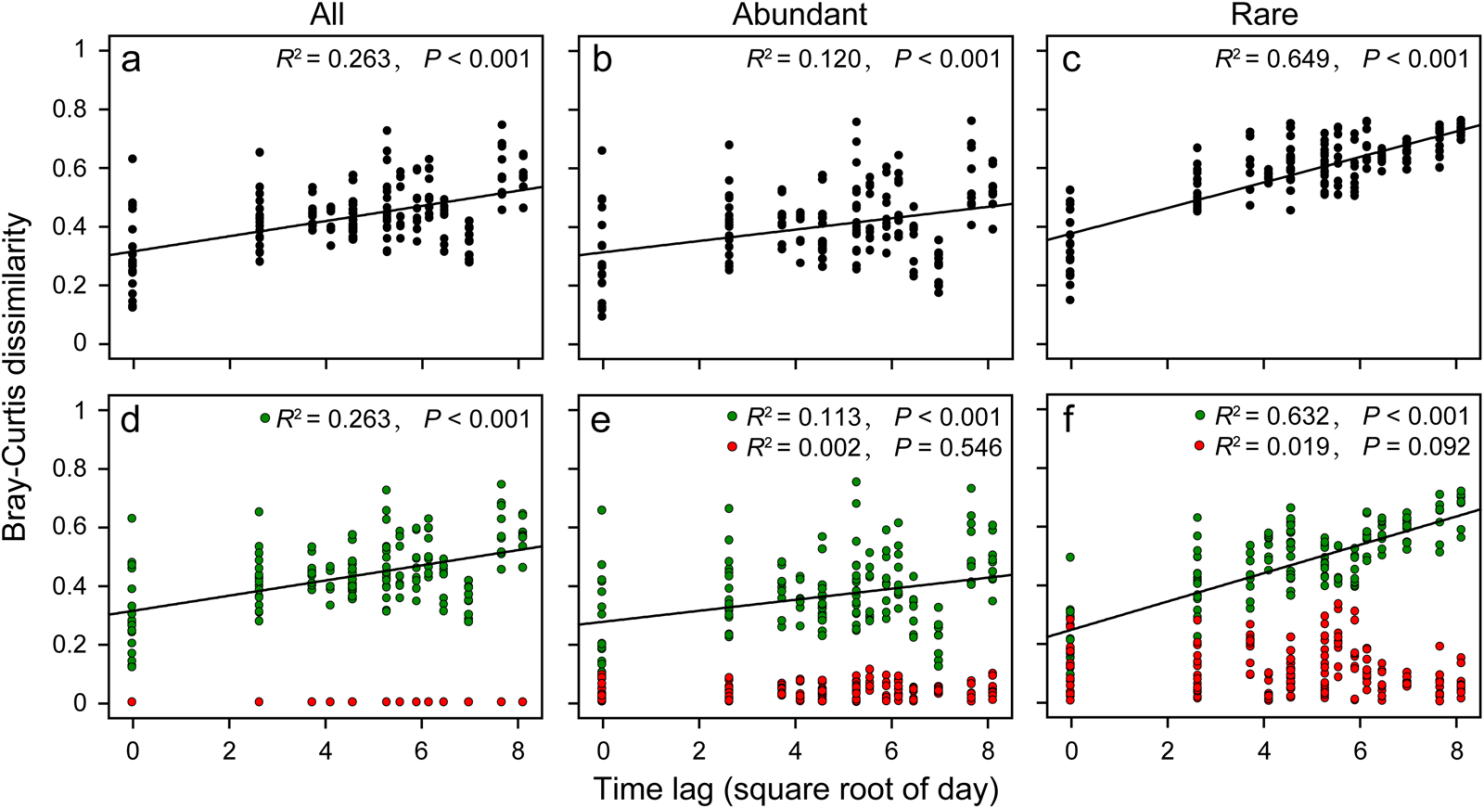
Time-lag regression analysis of total beta-diversity (**a**–**c**), and the turnover (green dots) and richness (red dots) components of beta-diversity (**d**–**f**) for all, abundant and rare eukaryotic plankton communities

Generally, the time-lag regression analysis had significant positive slopes, indicating both microbial plankton communities and environmental conditions were undergoing a directional change (Fig. 2a–c and Fig. S5). However, the slope for the rare taxa was significantly steeper than those in the all and abundant taxa. Beta-diversity partitioning further revealed that species replacement (turnover), rather than species richness, accounted for the majority of the beta-diversity, and drove the shift in community composition over time (Fig. 2d–f and Fig. S6).

Our results revealed that eukaryotic plankton communities clustered strongly by time rather than depth, and three groups of samples were clearly defined: bloom, post-bloom 1, and post-bloom 2 (Fig. 1a). The Mantel test results also demonstrated that eukaryotic plankton communities were mainly governed by temporal factors (Table 2). Rare taxa showed a striking separation compared with the all and abundant taxa, confirmed by the comparison of between-group distances among different periods and the results of ANOSIM analyses (Fig. 1c and Table 3). Further, abundant taxa exhibited greater niche breadth values than rare taxa among three periods (Fig. S7).

**Table 2:** Spearman’s correlations of the eukaryotic plankton community with environmental factors based on Mantel tests

Environmental factors	All	Abundant	Rare	Co-occurring network
Time	**0.334**	**0.222**	**0.652**	**0.334**
Depth	−0.037	−0.015	−0.120	−0.036
Temperature	**0.215**	**0.211**	**0.235**	**0.220**
Electrical conductivity	−0.121	−0.120	−0.106	−0.128
Turbidity	−0.094	−0.094	−0.062	−0.089
pH	0.100	0.070	**0.175**	0.111
Oxidation reduction potential	−0.132	−0.139	−0.027	−0.141
Dissolved oxygen	0.075	0.071	−0.039	0.069
Total carbon	**0.283**	**0.202**	**0.452**	**0.286**
Total organic carbon	**0.226**	0.193	**0.293**	**0.233**
Total nitrogen	−0.119	−0.121	−0.055	−0.130
Ammonium nitrogen	−0.039	−0.049	0.014	−0.051
Nitrate nitrogen	0.100	0.110	0.042	0.098
Nitrite nitrogen	**0.206**	0.167	**0.248**	**0.208**
Total phosphorus	−0.011	−0.018	0.035	−0.032
Phosphate phosphorus	−0.105	−0.073	−0.156	−0.121
Total nitrogen and total phosphorus ratio	**0.148**	0.117	**0.224**	0.136
Chlorophyll *a*	0.179	0.130	**0.311**	0.184

Time indicates three successional periods, including bloom, post-bloom 1, and post-bloom 2 periods

Depth denotes surface, middle, and bottom water layers

All, whole eukaryotic plankton communities; Abundant, abundant plankton subcommunities; Rare, rare plankton subcommunities

The significances are tested based on 999 permutations, and bold values indicate *P* < 0.05

**Table 3:** Analysis of similarity (ANOSIM) statistics testing differences of eukaryotic plankton community groupings at spatiotemporal scales

Grouping by	All		Abundant		Rare	
	*R*	*P*	*R*	*P*	*R*	*P*
Global time (month)	0.757	**0.001**	0.568	**0.001**	0.825	**0.001**
Bloom vs. Post-bloom 1	0.583	**0.002**	0.454	**0.002**	0.739	**0.002**
Post-bloom 1 vs. Post-bloom 2	0.656	**0.002**	0.485	**0.002**	0.761	**0.002**
Bloom vs. Post-bloom 2	0.989	**0.002**	0.713	**0.002**	0.996	**0.002**
Global space (depth)	−0.129	0.961	−0.115	0.944	−0.152	0.991
Surface vs. Middle	−0.087	0.729	−0.017	0.506	−0.144	0.903
Middle vs. Bottom	−0.172	0.972	−0.213	0.996	−0.159	0.937
Surface vs. Bottom	−0.120	0.851	−0.094	0.790	−0.152	0.939

An R-statistic less than 0 represents complete random grouping

All, whole eukaryotic plankton communities; Abundant, abundant plankton subcommunities; Rare, rare plankton subcommunities

Bold font indicates significant value (*P* < 0.01)

A greater number of taxonomic groups were present among rare planktonic eukaryotes than abundant ones (Fig. S8). The abundant taxa entailed only two major contributors to the community rearrangements, including Arthropoda (6.76%), for which the relative abundance of sequences declined over time, and Cryptophyceae (2.72%) characterized by a marked increase from bloom to post-bloom periods (Fig. S8c). By contrast, a large number of rare OTUs predominantly contributed to the dissimilarity in community composition. Altogether, they explained 65.76% of the total dissimilarity over time (Table S1). The main contributors to the rearrangement were unclassified eukaryotic groups (14.76%), Ciliophora (7.02%), Chrysophyceae (4.64%), Cryptophyceae (3.16%), which had the lowest abundance in post-bloom period 1, Chlorophyta (3.03%), Chytridiomycota (2.22%), which were particularly abundant during post-bloom period 2, and Cercozoa (3.11%), which were abundant during the bloom period (Fig. S8d).

### Co-occurrence networks of different subcommunities

**Fig. 3:**
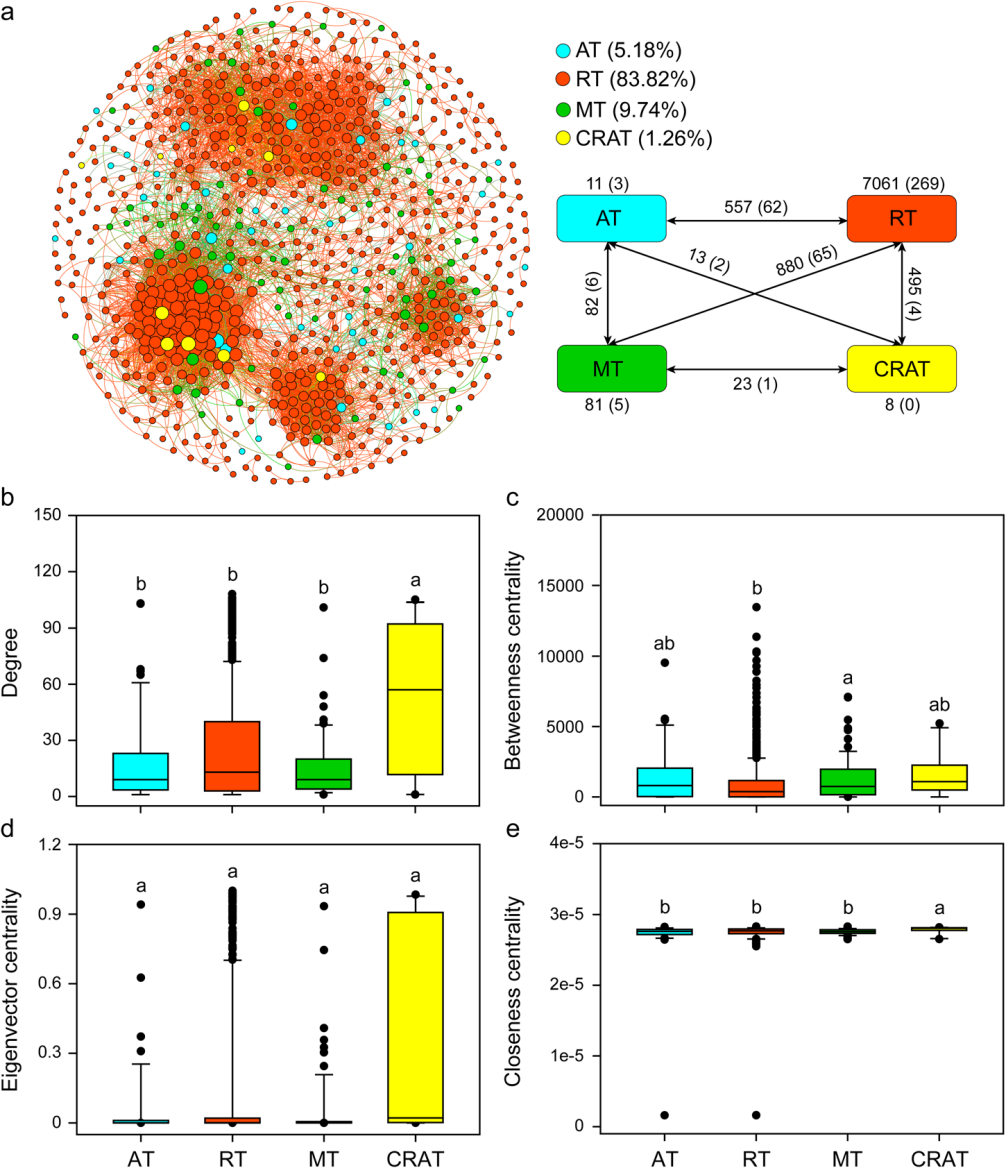
Properties of the correlation-based network. **a** The networks analysis showing the intra-associations within each subcommunity and inter-associations between different subcommunities. A connection stands for a strong (Spearman’s *r* > 0.8 or *r* < −0.8) and significant (*P*-value < 0.01) correlation. The size of each node is proportional to the number of connections (i.e., degree). Numbers outside and inside parentheses represent positive edge numbers and negative edge numbers, respectively. **b**–**e** Comparison of node-level topological features among four different subcommunities. The top and bottom boundaries of each box indicate the 75th and 25th quartile values, respectively, and lines within each box represent the median values. Different letters indicate the significant level at *P* < 0.05 level determined by nonparametric Mann–Whitney *U* test. AT abundant taxa, RT rare taxa, MT moderate taxa, CRAT conditionally rare and abundant taxa

A metacommunity co-occurrence network was built based on correlation relationships (Fig. 3a). The resulting network consisted of 791 nodes linked by 9628 edges (Table S5), with a much higher number of strong positive correlations observed than negative ones (Fig. 3a). The network obtained exhibited scale-free characteristics (power-law: *R*^2^ = 0.956, Fig. S9), indicating that the network structure was non-random. The observed modularity, average clustering coefficient and average path length were all greater than those of their respective Erdös–Réyni random networks, suggesting the network had “small-world” properties and modular structure (Table S5). Non-rare taxa (abundant, moderate, and conditionally rare and abundant taxa) frequently interacted more with rare taxa than with themselves (Fig. 3a).

We compared unique node-level topological features of four subcommunities. Both the degree and closeness centrality values of conditionally rare and abundant OTUs were highest among four subcommunities (Fig. 3b,e). Significantly lower betweenness centrality values were found for rare OTUs than for moderate taxa (Fig. 3c). However, eigenvector centrality values showed no significant differences between the four subcommunities (Fig. 3d). Keystone species, which play key roles in maintaining the structure and function of microbial communities, usually have high degree (>100) and low betweenness centrality values (<5000) in co-occurrence networks. Based on this criterion, a total of 17 OTUs were defined as keystone species, including Dinophyceae (1 OTU), Chlorophyta (2 OTUs), Cryptophyceae (2 OTUs), Cercozoa (2 OTUs), Chrysophyceae (3 OTUs), unidentified Stramenopiles (4 OTUs), and unclassified eukaryotic groups (3 OTUs), and all but two of these keystone species belonged to rare taxa (Table S6).

### Modular structure of the co-occurrence network

**Fig. 4:**
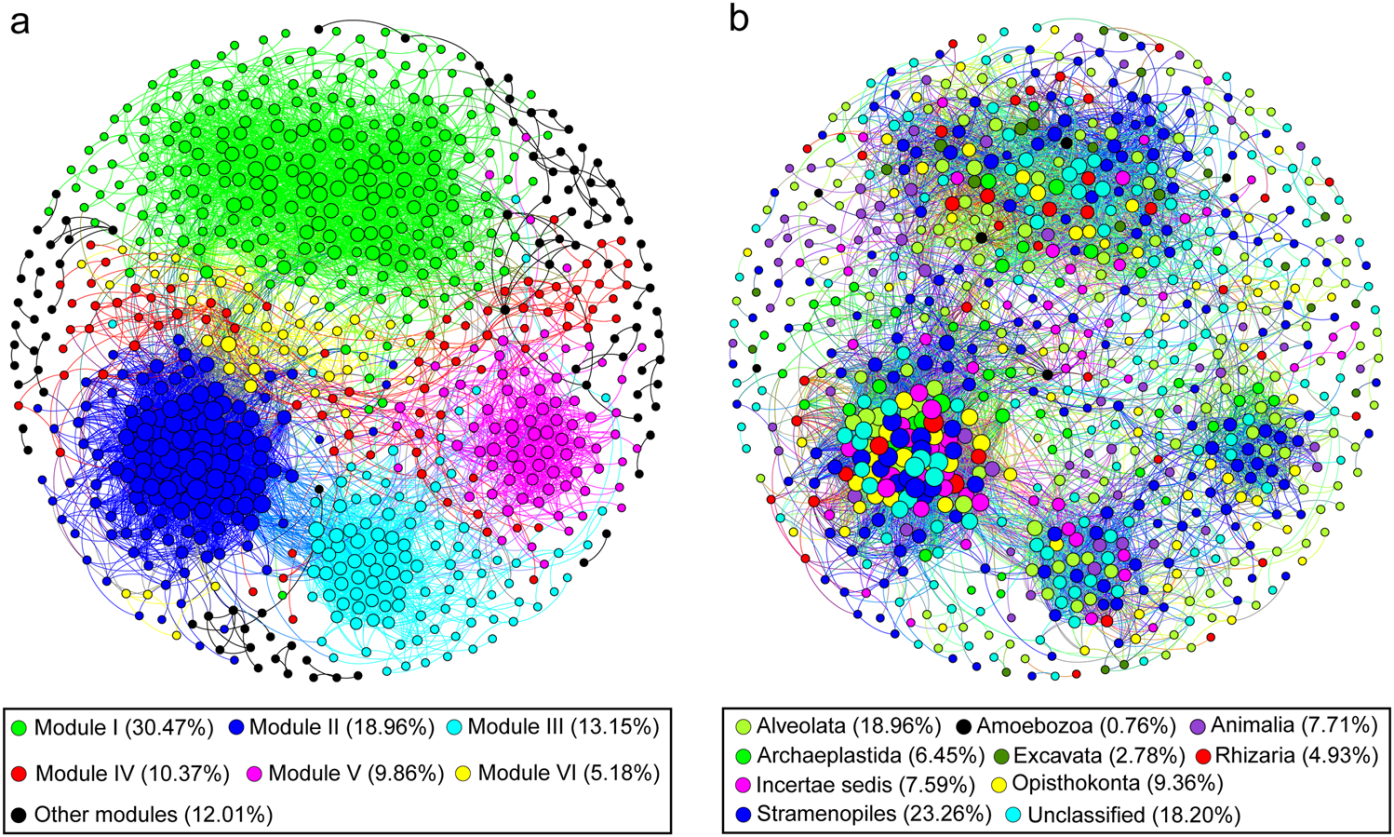
The co-occurrence patterns among OTUs revealed by network analysis. The nodes were colored according to different types of modularity classes (**a**) and supergroups (**b**), respectively. A connection stands for a strong (Spearman’s *r* > 0.8 or *r* < −0.8) and significant (*P*-value < 0.01) correlation. The size of each node is proportional to the number of connections (i.e., degree). Major modules have more than 40 nodes. Other modules include all small modules (*n* = 26) with nodes <18 per module

**Fig. 5:**
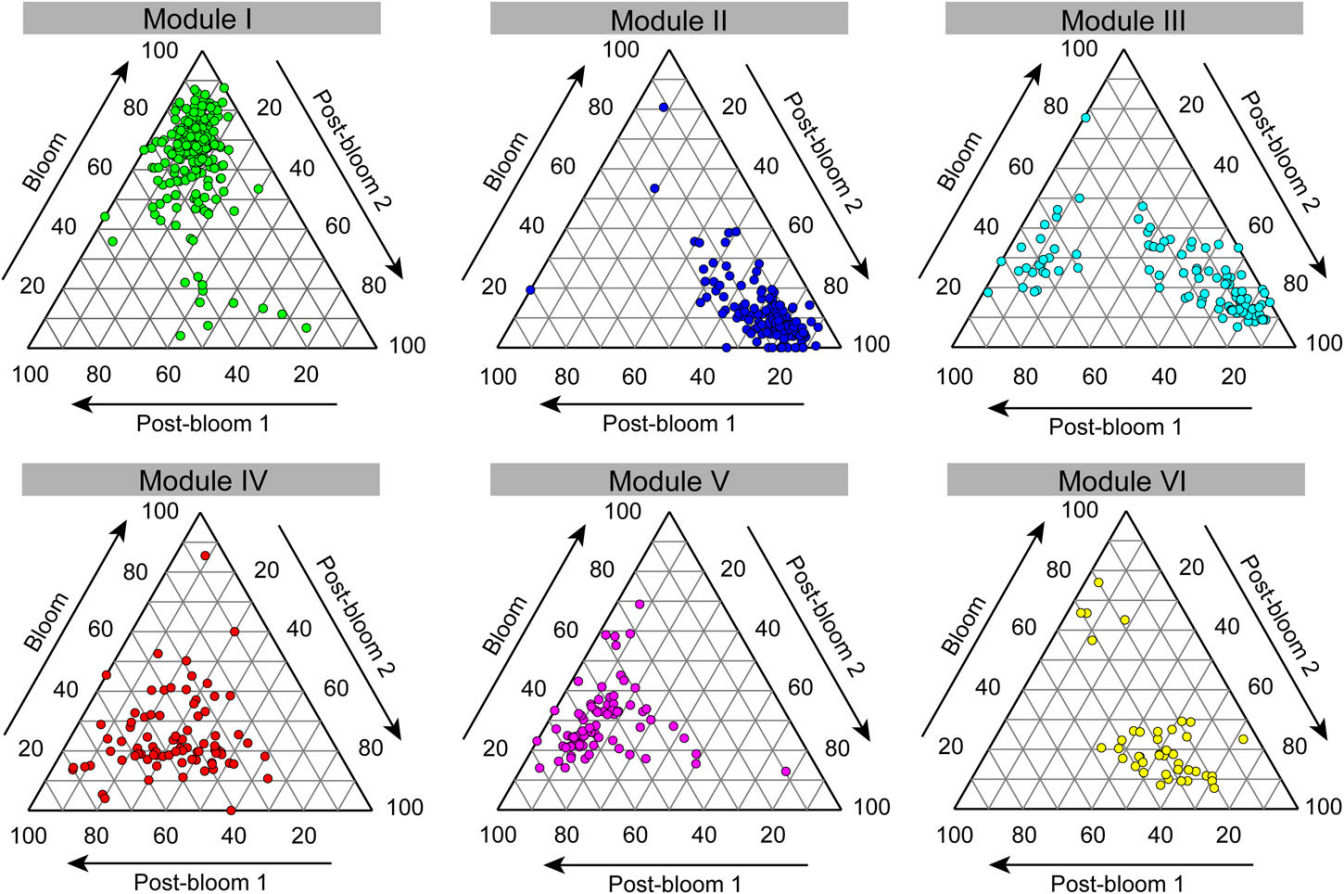
Ternary plots showing relative abundance of OTUs from modules I–VI in the three different periods. Each circle represents one individual OTU. For each OTU, abundance was averaged over all samples at each period

The entire network was clearly parsed into 6 major modules, of which modules I and II respectively accounted for 30.47 and 18.96% of the whole network (Fig. 4). Ternary plot analysis indicated that most modules were specific (relatively more abundant) to a particular period (Fig. 5). For example, most of the OTUs from the largest module I had higher relative abundances in the bloom period than post-bloom periods, whereas the majority of the OTUs from modules II, III, and VI had higher relative abundances in post-bloom period 2 than the other two periods. Importantly, taxonomic relatedness played a key role in determining the modular structure (Fig. 4a vs. Fig. 4b). The 6 major modules composed of substantial rare OTUs and few non-rare OTUs (abundant, moderate, and conditionally rare and abundant OTUs) were primarily occupied by Chrysophyceae, Cryptophyceae, Dinophyceae, Chlorophyta, Ciliophora, Cercozoa and Fungi (Fig. S10). Based on the values of connectivity, the co-occurrence network had 6 module hubs that belonged to rare taxa, and 12 OTUs were classified as connectors, including 2 abundant OTUs, 3 moderate OTUs, and 7 rare OTUs (Fig. S11). These module hubs and connectors primarily came from module I, IV, V, and VI (Table S7).

### Factors related to variation of the eukaryotic plankton community

The Mantel test results indicated that the changes of all, rare and network communities were correlated with temperature, total carbon (TC), total organic carbon (TOC), and nitrite nitrogen (NO_2_-N) (Table 2). In addition, pH, total nitrogen and total phosphorus ratio (TN:TP), and chlorophyll *a* (Chl-*a*) were also significantly related to the variation of rare community composition. In contrast to rare taxa, only temperature and TC were significantly correlated with the abundant taxa. More interestingly, the neutral community model successfully explained a large fraction of variation in both all (*R*^2^ = 0.895) and rare (*R*^2^ = 0.879) plankton communities (Fig. S12).

To investigate the modules’ responses to environmental conditions, significant edge numbers between environmental factors and OTUs from each module were calculated (Spearman’s *r* > 0.8 or *r* < −0.8, and *P*-value < 0.01). Among environmental factors, we found that temperature, TC, TOC, NO_2_-N, and Chl-*a* were frequent drivers of network connections (Fig. S13).

## Discussion

Eukaryotic plankton in freshwater ecosystems are considered to be one of the indicators of environmental change and ecosystem state owing to their fast and strong responses to environmental disturbances [2, 23]. We found that abundant and rare eukaryotic plankton communities exhibited different and complex responses to environmental changes. Therefore, elucidating the mechanisms of abundant and rare eukaryotic succession under changing environments is crucial for understanding the process of ecosystem recovery from disturbance events (e.g., cyanobacterial blooms).

### Dynamics of abundant and rare eukaryotic plankton communities

Our results showed that the effect of the cyanobacterial bloom was detectable by its influence on the composition of the eukaryotic plankton community (Fig. 1). The similarity between samples significantly decreased with the increase in time-lag, illustrating that eukaryotic plankton communities were not resilient and had reached an alternative state (Fig. 2a-c). Usually, resilience is defined as the degree to which a system returns to the original state after a perturbation [49]. However, resilience can also be described as the recovery process to an alternative stable state following a disturbance [2]. More importantly, our results indicated that different taxa did not respond equally to the cyanobacterial bloom. Rare taxa showed greater differences in both diversity and community composition between three periods than abundant taxa (Tables 1 and 3), and the temporal turnover of rare taxa (*R*^2^ = 0.649, *P* < 0.001) was significantly higher than that of the abundant ones (*R*^2^ = 0.120, *P* < 0.001) (Fig. 2b,c). These results indicated that the stable predominance of a few highly abundant taxa is contrasted by a highly dynamic turnover of rare species, as previously observed in Austrian lakes [50].

We found that the cyanobacterial bloom event significantly altered the eukaryotic plankton community composition without affecting overall diversity. This may reflect the fact that microorganisms usually have a high population density, and more individuals with versatile physiology contributing to the high resistance in biodiversity [51]. The rare subcommunities had ~50 times higher richness than the abundant subcommunities, supporting the idea that rare microbes are important contributors to microbial diversity [19]. Meanwhile, the diversity indices of rare subcommunities exhibited temporal differences, but the abundant subcommunities had no significant difference among three periods. This may be due to the immigration and emigration of rare species and the recovery of dormant taxa [29]. Due to their high diversity, rare populations could increase the functional redundancy of the community [52], further providing biological buffering capacity to withstand environmental changes [53].

### Controlling mechanisms and factors shaping the eukaryotic plankton community

A central challenge in ecology is to quantify the relative roles of deterministic and stochastic processes that shape the assembly of microbial communities [54]. Niche theory holds that microbial communities are shaped by deterministic processes (e.g., habitat heterogeneity or species sorting) owing to different habitat preferences and fitness of species, whereas according to neutral theory, microbial communities are shaped by random fluctuations in species abundance (birth and death) and limited dispersal [55]. Both deterministic and stochastic processes can act concurrently to regulate the assembly of microbial communities [12]. In this study, water temperature, TC, TOC, NO_2_-N and TN:TP were significantly related to the temporal variability of eukaryotic community composition (Table 2). These relationships have also been reported in previous studies of eukaryotic plankton communities from both a shallow lake and a subtropical river [25, 56]. As expected, rare taxa were more sensitive to environmental filtering compared with abundant taxa. It is possible that abundant taxa competitively utilize a broad array of resources and are well adapted to a particular ecosystem through active growth [57], whereas rare taxa have less competition capability and intrinsically low growth rates [58], thereby being restricted to fewer samples. This suggests that environmentally-induced species sorting has a strong impact on the composition of microbial communities [59]. Furthermore, abundant taxa had wider temporal niche breadths than rare ones, and a significant positive abundance-occurrence relationship was observed (Fig. S7). The analysis of abundance-occurrence can be a highly valuable approach for distinguishing where shifts in microbial communities occur across environmental gradients. Our samples spanned contrasting environmental conditions, where continuous environmental change resulted in the existence of multiple niches. A total of 131 strict indicator OTUs were found across three different periods, and the majority of strict specialists were affiliated to rare taxa (Table S4). This feature suggests that the occurrence and disappearance of cyanobacterial blooms can induce environmental heterogeneity, with local habitat conditions selecting for specific taxonomic groups.

We also found that stochastic processes may play an important role in eukaryotic plankton community assembly. In our study, rare taxa gave a good fit to the neutral model, and over 80% of community turnover for rare taxa was well explained by stochastic processes (Fig. S12). By investigating the distribution of abundant and rare estuarine fish communities over 21 years, Magurran and Henderson [60] found that abundant species associated with estuarine habitats were log normally distributed, while rare species had different habitat requirements and followed a log series distribution. The stochastic pattern was particularly pronounced for the rare taxa, and so overall the plankton data set was a good fit to the neutral model (*R*^2^ = 0.895, Fig. S12). Moreover, the proportion of turnover partitioning in the beta-diversity for rare taxa was higher than that of abundant ones (Fig. S6), suggesting that rare taxa with a low abundance are more likely to be lost due to ecological drift (i.e., the stochastic loss and replacement of individuals) [61]. Overall, both deterministic and stochastic processes appear to significantly influence the temporal dynamics of eukaryotic plankton community composition. Recent studies showed that two types of processes, deterministic and stochastic, influence the microbial community assembly with varying relative effects depending on geographic scales and strength of environmental gradients [54, 62]. In aquatic ecosystems, cyanobacterial blooms can generate sequential changes both in the overall planktonic structure and in environmental conditions [4]. Future studies should pay more attention to whether the strength of ecological selection and rates of dispersal vary with different cyanobacterial states (e.g., bloom vs. non-bloom) with large sample size, thereby providing directions to better understand the mechanisms governing the balance between deterministic and stochastic processes in plankton succession under changing conditions.

### Effect of interspecies interactions on the dynamics of eukaryotic plankton community

Network analysis can potentially provide deep and unique perspectives on microbial interactions and ecological assembly rules beyond those of simple richness and composition [63]. Here, for the first time, we applied correlation-based network analysis to explore the co-occurrence patterns of abundant and rare eukaryotic plankton communities cross a cyanobacterial bloom event. The resulting plankton network had statistical and structural characteristics similar to those of bacterial ecological networks [28, 64], such as non-randomly connected properties, power-law distribution, and modular structure. The topology of the networks can reflect interactions between microorganisms. For example, the degree value describes the level of connectedness between OTUs, and the betweenness centrality value provides information on how critical an OTU is to the connectedness of a network [45]. Our results showed that rare OTUs had higher degree value but a lower betweenness centrality value than abundant OTUs, but the differences were not significant. According to the generation process of a scale-free topology, keystone nodes are commonly recognized as initiating components in networks [65], which tend to have high degree and low betweenness centrality values [66]. All keystone species in our co-occurrence networks were affiliated to rare taxa with the exception of a conditionally abundant OTU and a conditionally rare and abundant OTU. This suggests that rare species may play an irreplaceable role in maintaining the structure of microbial communities. In a co-occurrence network, the positive interaction is mainly regarded as cooperation [67]. We found that rare taxa showed more positive interactions to non-rare taxa (abundant, moderate, and conditionally rare and abundant taxa) than negative ones. Interactions between microorganisms can support ecosystem function and stability, for instance, rare *Symbiodinium* taxa can cause a significant increase in the stability of the coral-symbiont community under environmental changes [63]. Similarly, the cooperation between rare and non-rare taxa might contribute to the resilience of the microbial plankton community under a changing environment, because the interaction network of plankton can provide a buffer against the environmental disturbance [49]. On the other hand, species played different roles with respect to modularity. In our study, all 6 module hubs (i.e., nodes highly linked within their own module) and 7 out of 12 connectors (i.e., nodes linking different modules together) belonged to rare OTUs. Shi et al. [68] recently reported that less-abundant taxa can act as important keystone taxa in the rhizosphere networks. The disappearance of these key species may cause modules and networks to break apart [46], and thus rare species can be as important as or more important than the abundant ones in maintaining ecosystem stability.

By comparing the taxonomy of OTU nodes with the network modular structure, we found that the assembly of the eukaryotic plankton community was non-randomly determined by taxonomic relatedness, that is, closely related taxa tended to be highly interconnected and clustered together. This non-random pattern was evident in our network modules, because most modules exhibited a distinct temporal variation. Modularity may reflect synergistic relations, competitive interactions, and niche differentiation, leading to non-random patterns of interaction and ultimately contributing to the complexity of ecological networks [69]. The division of the network into modules may shed light on the different groups of nodes performing different functions [70]. For instance, module I was dominated by heterotrophic Ciliophora and Cercozoa, which are important consumers of picophytoplankton and bacteria [71, 72]. Phytoplankton blooms can boost the rate of bacterial growth and production [73]. Therefore, the increase in the abundance of these taxa is most likely derived from the increase in the abundance of their prey or food. Module II, III, and VI were specific to the post-bloom period 2 and were phototroph-dominated (e.g., Chrysophyceae and Cryptophyceae). Cryptophyceae was previously found to be favored by reductions of nutrient loading in shallow lakes [74], providing evidence for the existence of distinct ecological niches over temporal scales in the reservoir ecosystem in tandem with the disappearance of cyanobacterial bloom. In this study, rare species were widely located in all modules as central hubs, linking diverse abundant, moderate, and conditionally rare and abundant taxa. Rare microbes sustain a vast functional gene pool and can indirectly enhance functionality of abundant microbes [20]. Similarly, the interactions between rare taxa and non-rare taxa (abundant, moderate, and conditionally rare and abundant taxa) may affect biogeochemical cycles. For example, ciliates grazing upon picophytoplankton and bacteria likely play an essential role in transferring carbon and nutrients to higher trophic levels [75]. In both the modules I and II, ciliates showed more interactions with Chlorophyta and total (organic) carbon (Fig. S13). Parasitic fungal chytrids are common parasites of phytoplankton (e.g., colonial cyanobacteria and diatoms), which had an important influence on food web dynamics [76]. In the module II, the change in Chytridiomycota abundance was concomitant with the change in the abundance of Diatomea (Fig. S13). Such a synchronous change can affect the transfer of carbon from primary production to higher trophic levels [77]. Moreover, we found total (organic) carbon was significantly related to eukaryotic plankton dynamics (Fig. S13). Through these results, network analysis exhibited potential importance in unraveling the intrinsic mechanisms of interspecific interactions and understanding the roles of community members in ecological processes like carbon dynamics. However, in addition to considering species interactions based on the statistical and structural features of networks, both microbial parasites (e.g., chytrid parasitism on cyanobacteria) and interspecific allelopathy in cyanobacteria based on experiments and models can have large influences on food web structure and function [76, 78], and are essential for a good diagnosis, thereby providing directions for future ecology studies on eukaryotic plankton during cyanobacterial blooms.

### Conclusions and implications

Our results clearly demonstrated that the community composition of rare eukaryotic plankton had a stronger temporal pattern than that of abundant taxa following a cyanobacterial bloom. Due to high levels of dispersal, competition for resources and growth rates, the temporal stability of abundant taxa was greater than that of rare taxa. Both deterministic and stochastic processes simultaneously affected the community assembly of eukaryotic plankton, and random patterns (e.g., ecological drift) were particularly pronounced for the rare taxa. These results expanded our knowledge of the temporal patterns and ecological processes behind changes in the plankton community in a changing environment with emphasize on rare subcommunities. Co-occurrence network analysis further revealed that the synergistic effects between rare and non-rare taxa may play central roles in maintaining the stability of eukaryotic community and ecological function (e.g., carbon transfer or flow within the ecosystem). Additionally, eukaryotic plankton networks followed the same principles as bacterial communities, such as power-law distribution, non-randomly connected properties, module structure and “small-world” properties. In summary, our results provide a new perspective for the ecological significance of rare eukaryotic plankton in aquatic ecosystems, reinforcing recent ideas about microbial interactions among eukaryotic plankton.

## Electronic supplementary material

Supplementary information
