# Supplementary material for: Distinct patterns and processes of abundant and rare eukaryotic plankton communities following a reservoir cyanobacterial bloom
Source: ISME J. 2018 Jun 13;12(9):2263–77. doi: 10.1038/s41396-018-0159-0 (PMC6092360; doi:10.1038/s41396-018-0159-0)
Supplement: Supplementary file 1 — Supplementary information [file 41396_2018_159_MOESM1_ESM.doc]

**Journal: The ISME Journal**

*Supplementary information of the article:*

**Distinct patterns and processes of abundant and rare** **eukaryotic plankton communities following a reservoir cyanobacterial bloom**

Yuanyuan Xue^1,2,3^, Huihuang Chen^1^, Jun R. Yang^1,2^, Min Liu^1,2,3^, Bangqin Huang^3^ and Jun Yang^1^,*

^1^ Aquatic EcoHealth Group, Key Laboratory of Urban Environment and Health, Institute of Urban Environment, Chinese Academy of Sciences, Xiamen 361021, China

^2^ University of Chinese Academy of Sciences, Beijing 100049, China

^3^ College of Environment and Ecology, Xiamen University, Xiamen 361102, China

**Running title:** Abundant and rare eukaryotic plankton dynamics

**Subject category:** Microbial population and community ecology

***Correspondence:** Jun Yang, E-mail: jyang@iue.ac.cn

**This supplementary information contains:**

- 26 Pages
- 13 Figures
- 7 Tables
- 7 References


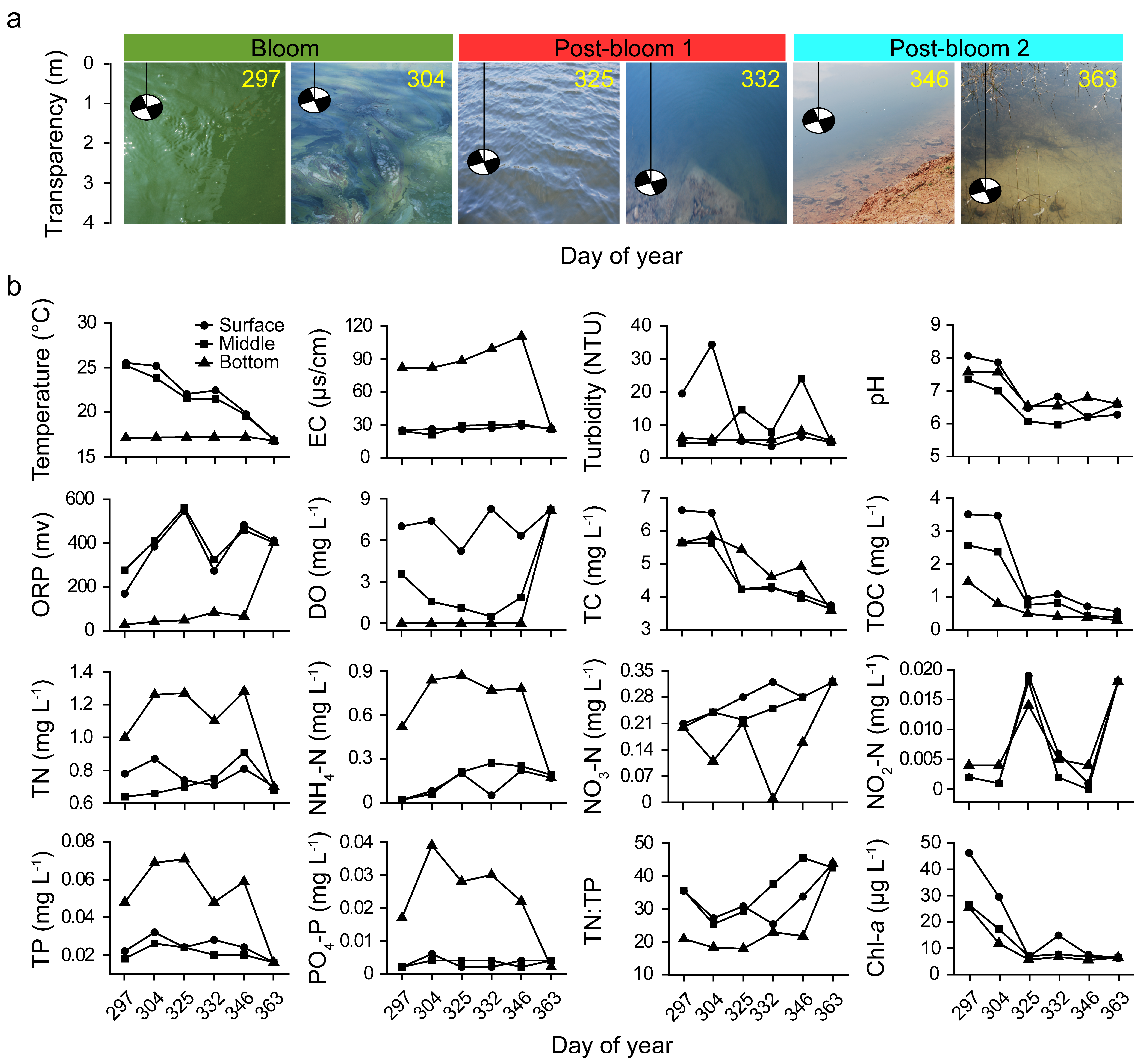


**Fig. S1** Dynamics of environmental parameters in Xidong Reservoir during the studied period. **a** Water transparency from cyanobacterial bloom to post-bloom periods, revised from Xue et al. [1]. **b** Spatial and temporal dynamics of 16 environmental parameters. EC electrical conductivity, ORP oxidation reduction potential, DO dissolved oxygen, TC total carbon, TOC total organic carbon, TN total nitrogen, NH_4_-N ammonium nitrogen, NO_3_-N nitrate nitrogen, NO_2_-N nitrite nitrogen, TP total phosphorus, PO_4_-P phosphate phosphorus, TN:TP total nitrogen and total phosphorus ratio, Chl-*a* chlorophyll *a*.


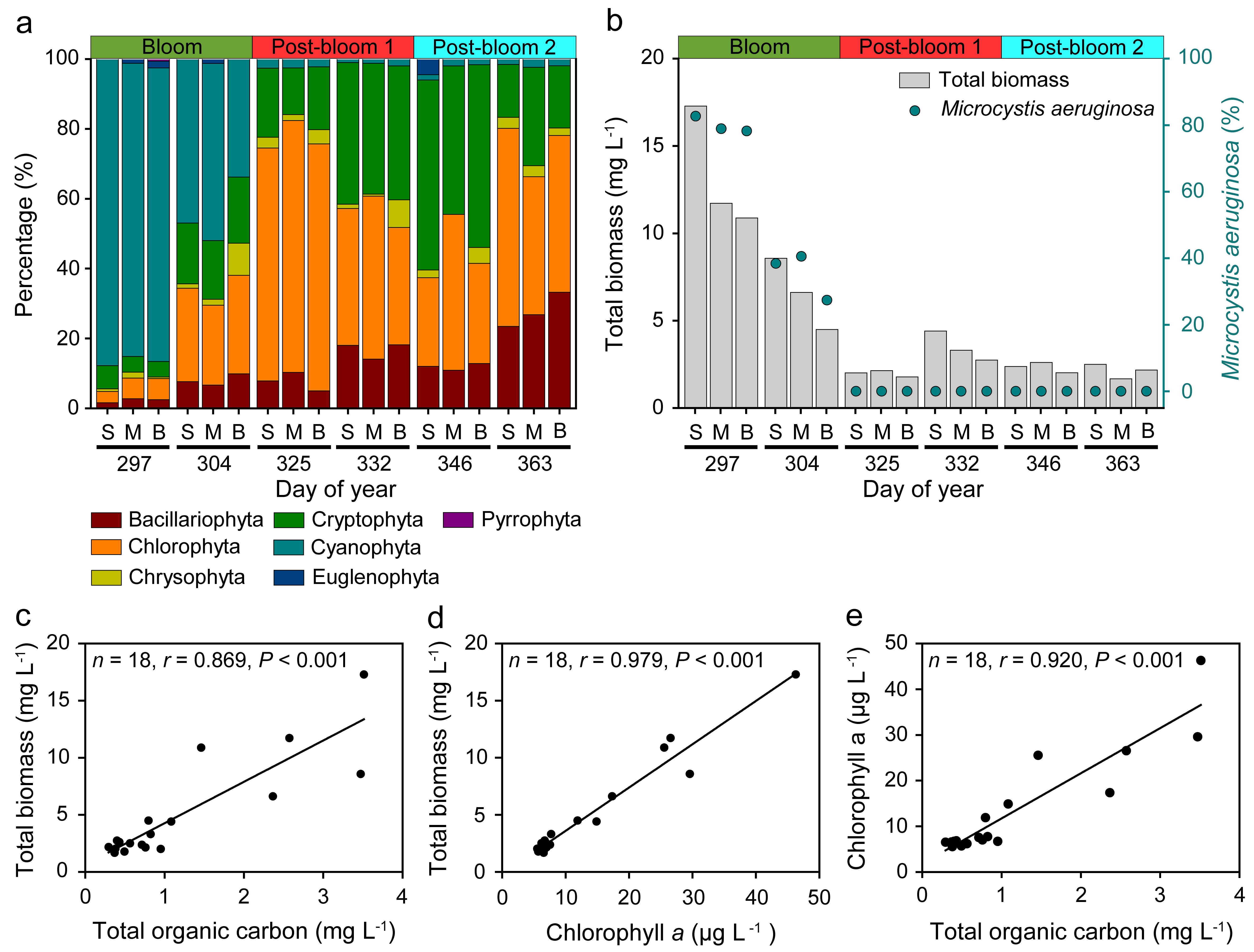


**Fig. S2** Phytoplankton community characteristics of Xidong Reservoir during the studied period. **a** Phytoplankton community succession of major taxonomic groups from cyanobacterial bloom to post-bloom periods. **b** Temporal patterns of total phytoplankton biomass and relative biomass of *Microcystis aeruginosa*, showing higher percentage of *Microcystis aeruginosa* during the bloom than post-bloom periods. The letters S, M and B denote surface, middle, and bottom water layers, respectively. **c** Correlation between total phytoplankton biomass and total organic carbon. **d** Correlation between total phytoplankton biomass and chlorophyll *a*. **e** Relationship between chlorophyll *a* and total organic carbon. Phytoplankton were identified and counted using an inverted microscope [2]. Temporal patterns of the phytoplankton community can be divided into two stages throughout the water column: the cyanobacteria dominant phase during the bloom period and non-cyanobacteria taxa dominant phase during the post-bloom periods. The dominant cyanobacteria during the bloom period was *Microcystis aeruginosa*.

**
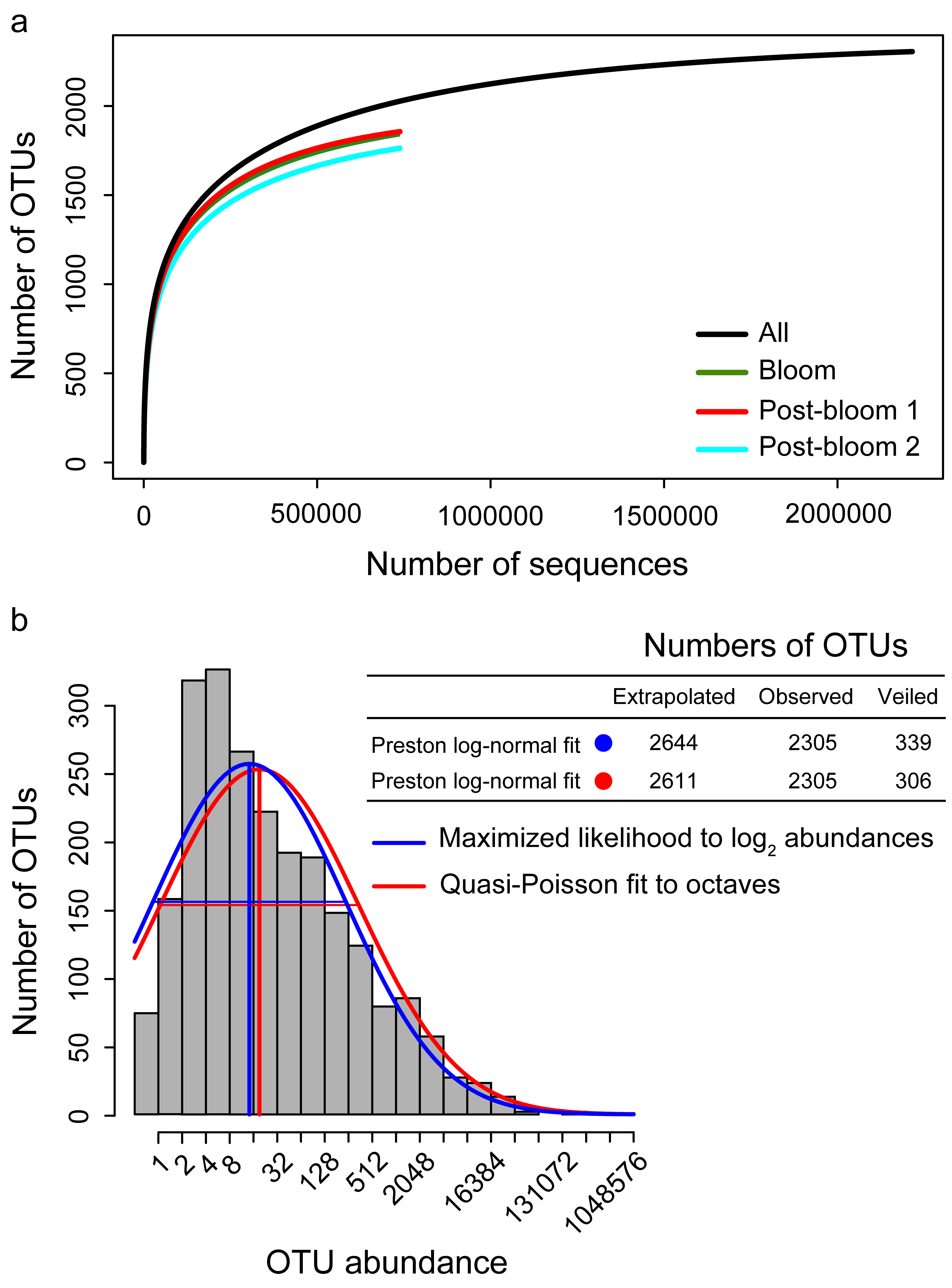
**

**Fig. S3** Eukaryotic plankton diversity of Xidong Reservoir. **a** Rarefaction curves of similarity-based operational taxonomic unit (OTU) at 97% sequence similarity level. **b** OTU abundance distribution and fit to the Preston log-normal model using two approximations: maximized likelihood to log_2_ abundances (blue line) and Quasi-Poisson fit to octaves (red line). Calculation of the Preston veil, which infers the number of OTUs that we missed during our sampling, confirmed that we captured most of the eukaryotic richness, thus allowing extraction of general patterns of eukaryotic plankton biodiversity from our data set.


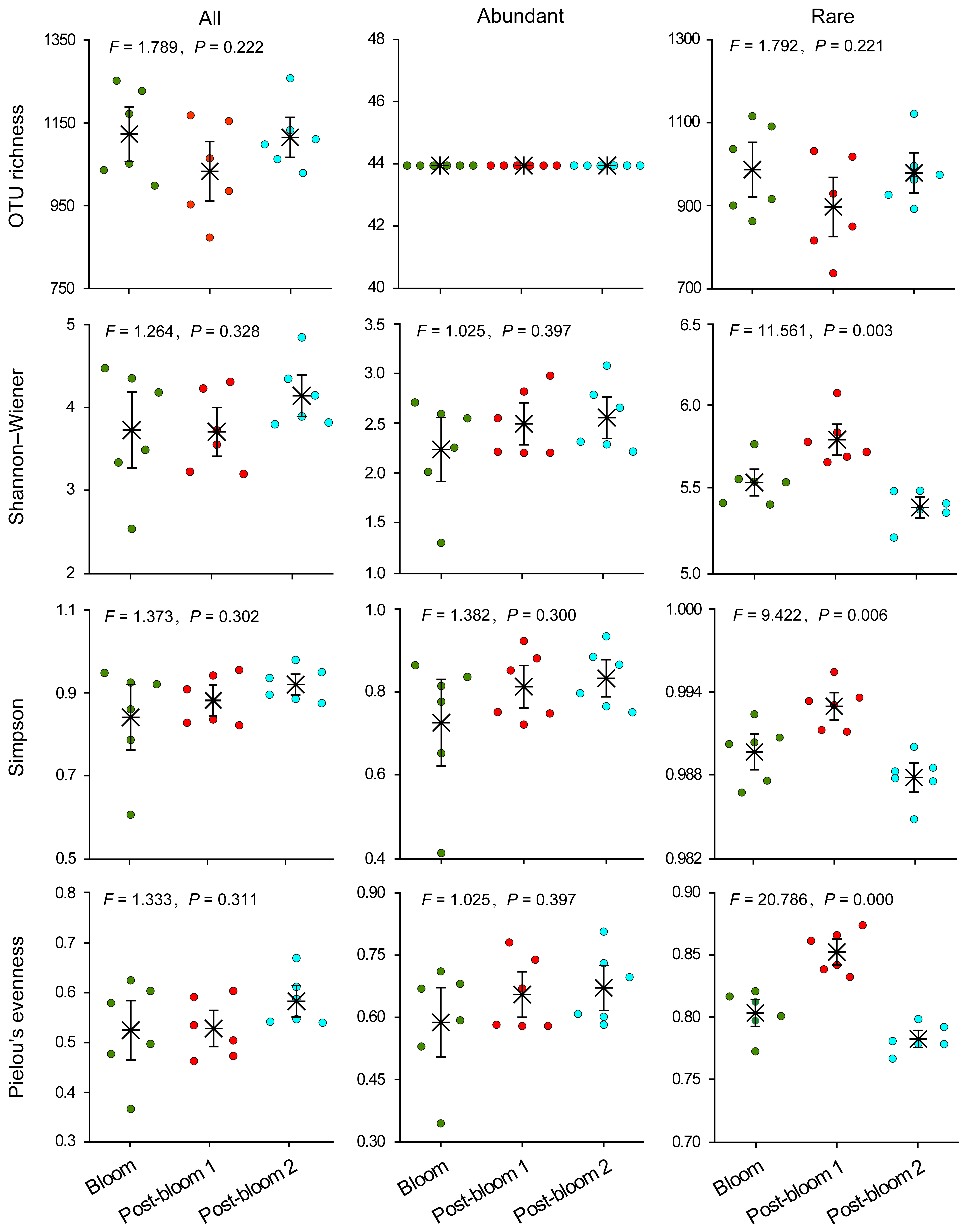


**Fig. S4** Variation in alpha-diversity through time. One-way ANOVA was used to compare the differences among different periods. Asterisks represent mean values (*n* = 6), and error bars are standard error. Note that abundant OTUs were persistent across all samples, and the *F* and *P* values of richness cannot be calculated. All, whole eukaryotic plankton; Abundant, abundant eukaryotic plankton; Rare, rare eukaryotic plankton.


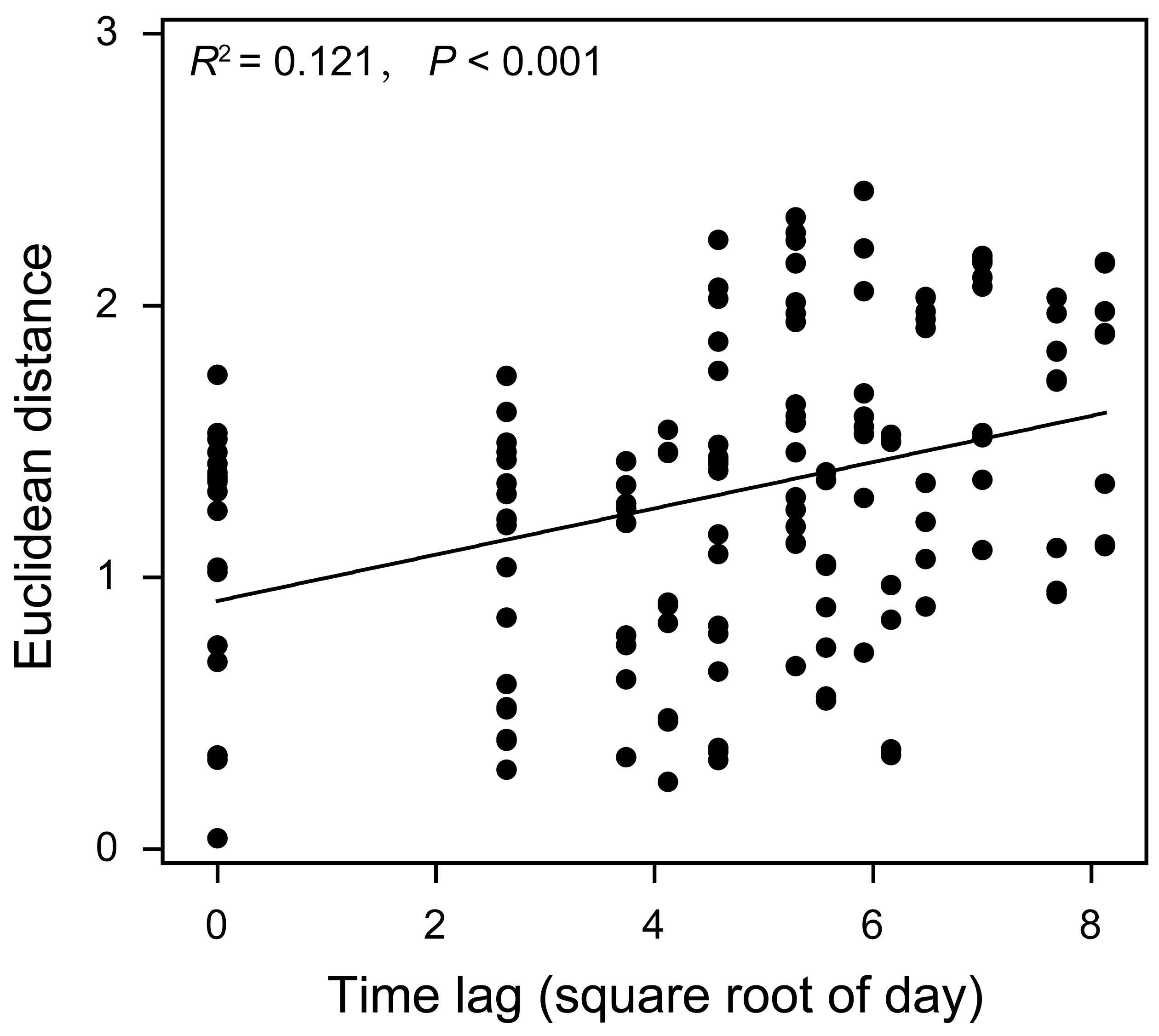


**Fig. S5** Time-lag regression analysis of changes in environmental variables (*n* = 153). Environmental variables investigated in the regression analysis were water temperature, electrical conductivity, turbidity, pH, oxidation reduction potential, dissolved oxygen, total carbon, total organic carbon, total nitrogen, ammonium nitrogen, nitrate nitrogen, nitrite nitrogen, total phosphorus, phosphate phosphorus, total nitrogen and total phosphorus ratio and chlorophyll *a*.


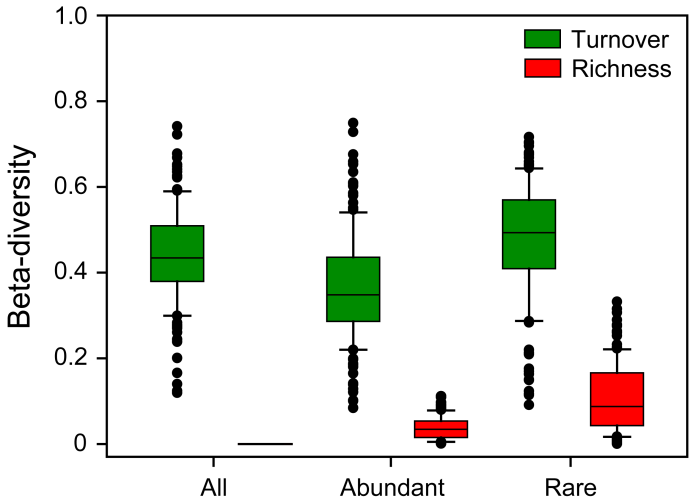


**Fig. S6** The contributions of turnover and richness components to the beta-diversity of all, abundant and rare eukaryotic plankton communities, respectively. The top and bottom boundaries of each box indicate the 75th and 25th quartile values, respectively, and lines within each box represent the median values (*n* = 153).


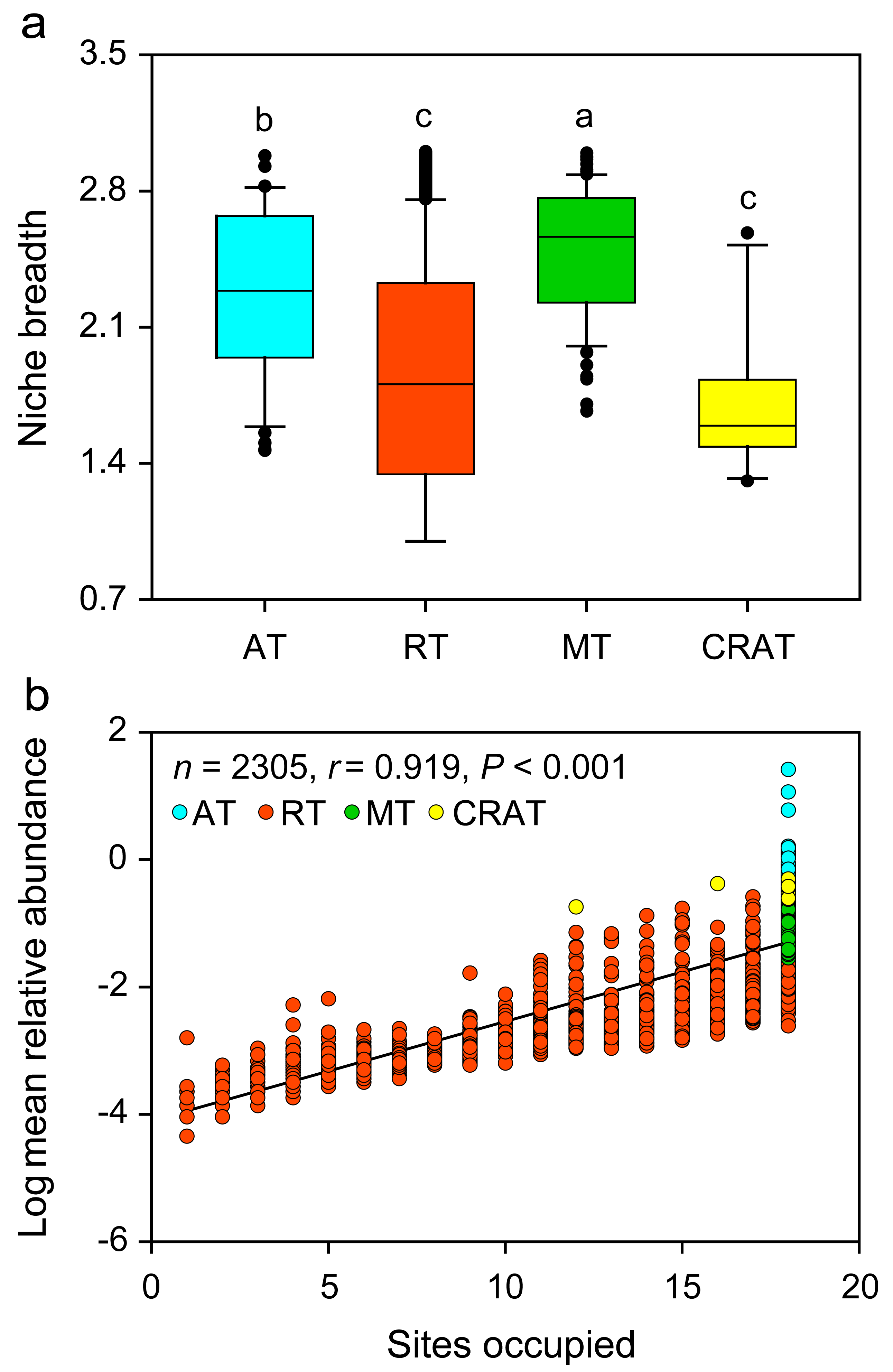


**Fig. S7** Occurrence and abundance of eukaryotic plankton. **a** Boxplots showing the mean niche breadth for four categories of eukaryotic plankton. Different letters indicate the significant level at 5% determined by nonparametric Mann-Whitney *U* test. The top and bottom boundaries of each box indicate the 75th and 25th quartile values, respectively, and lines within each box represent the median values (*n* = 44 for AT, *n* = 2167 for RT, *n* = 84 for MT, and *n* = 10 for CRAT). **b** Spearman’s rank correlation between the relative abundance of eukaryotic plankton OTUs and number of sites occupied (*n* is the number of OTUs). AT abundant taxa, RT rare taxa, MT moderate taxa, CRAT conditionally rare and abundant taxa.


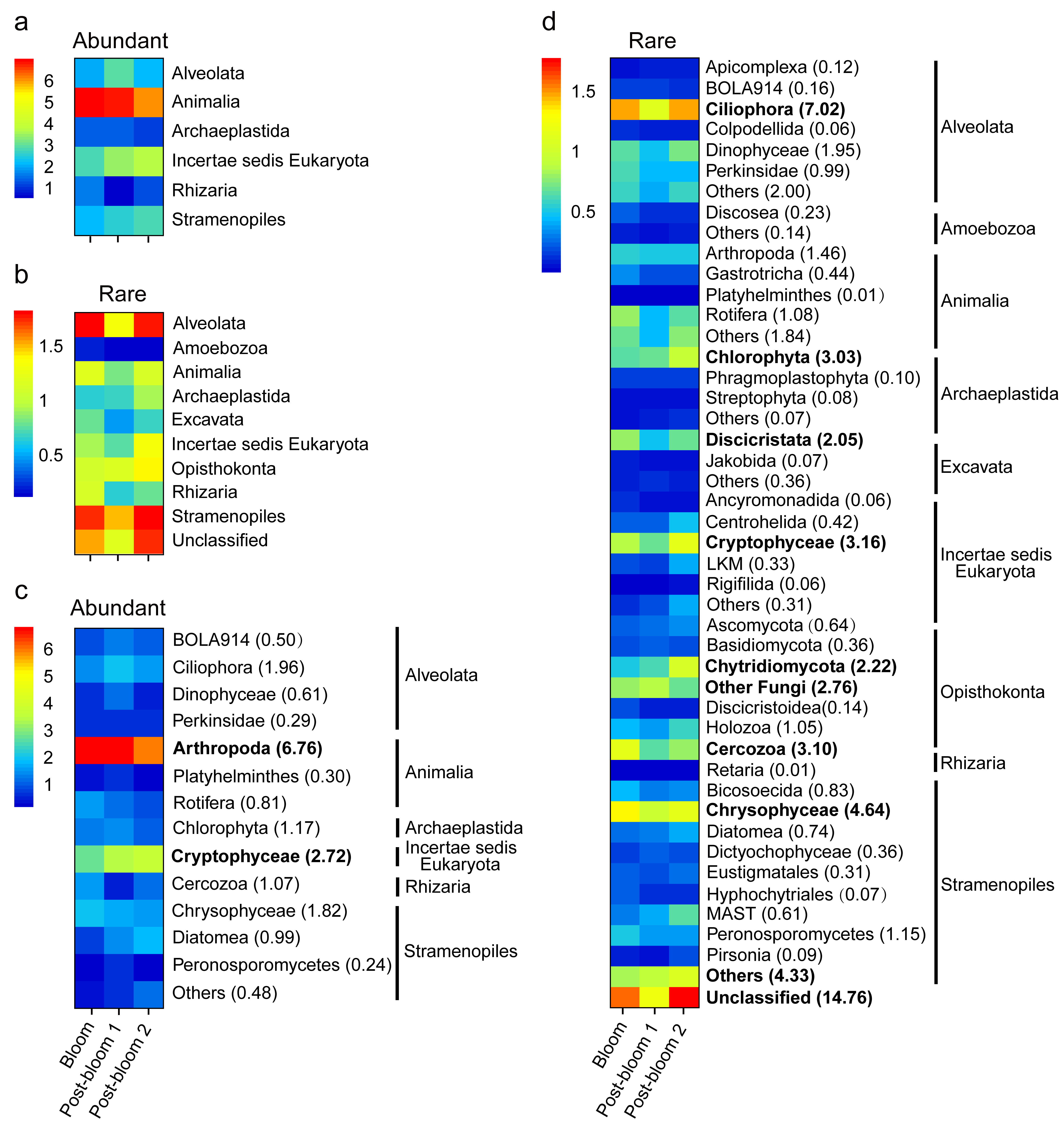


**Fig. S8** Comparison of community composition of abundant and rare eukaryotic plankton among different periods at the super-group level (**a, b**) and at the phylum or class level (**c, d**). Numbers in parentheses refer to the contribution of each taxonomic group to the community dissimilarity across three consecutive periods. The considered periods are those defined by ANOSIM analysis, including bloom, post-bloom 1 and post-bloom 2 periods. Each cell represents the square root transformed mean relative abundance of each taxonomic group at each period. Others in each super-group include the rare lineages (OTUs < 3 and sequences < 100) and unidentified lineages. Abundant, abundant taxa; Rare, rare taxa.


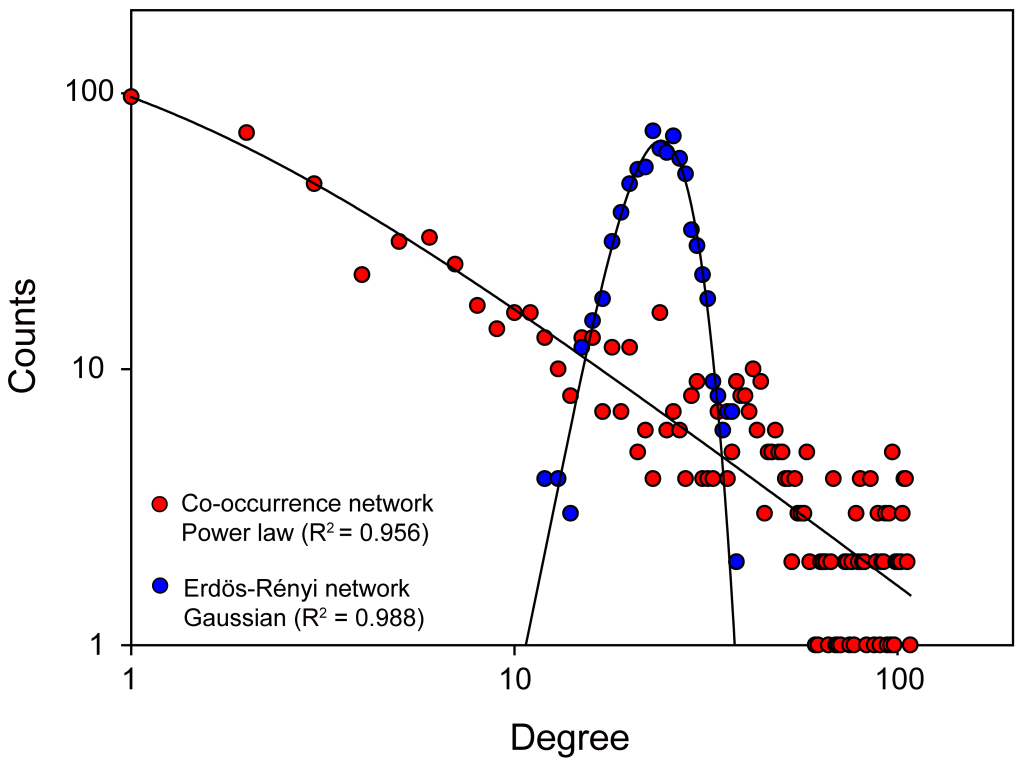


**Fig. S9** The distributions of degree for the real co-occurrence network (red) and Erdös-Rényi random network (blue) of eukaryotic plankton.


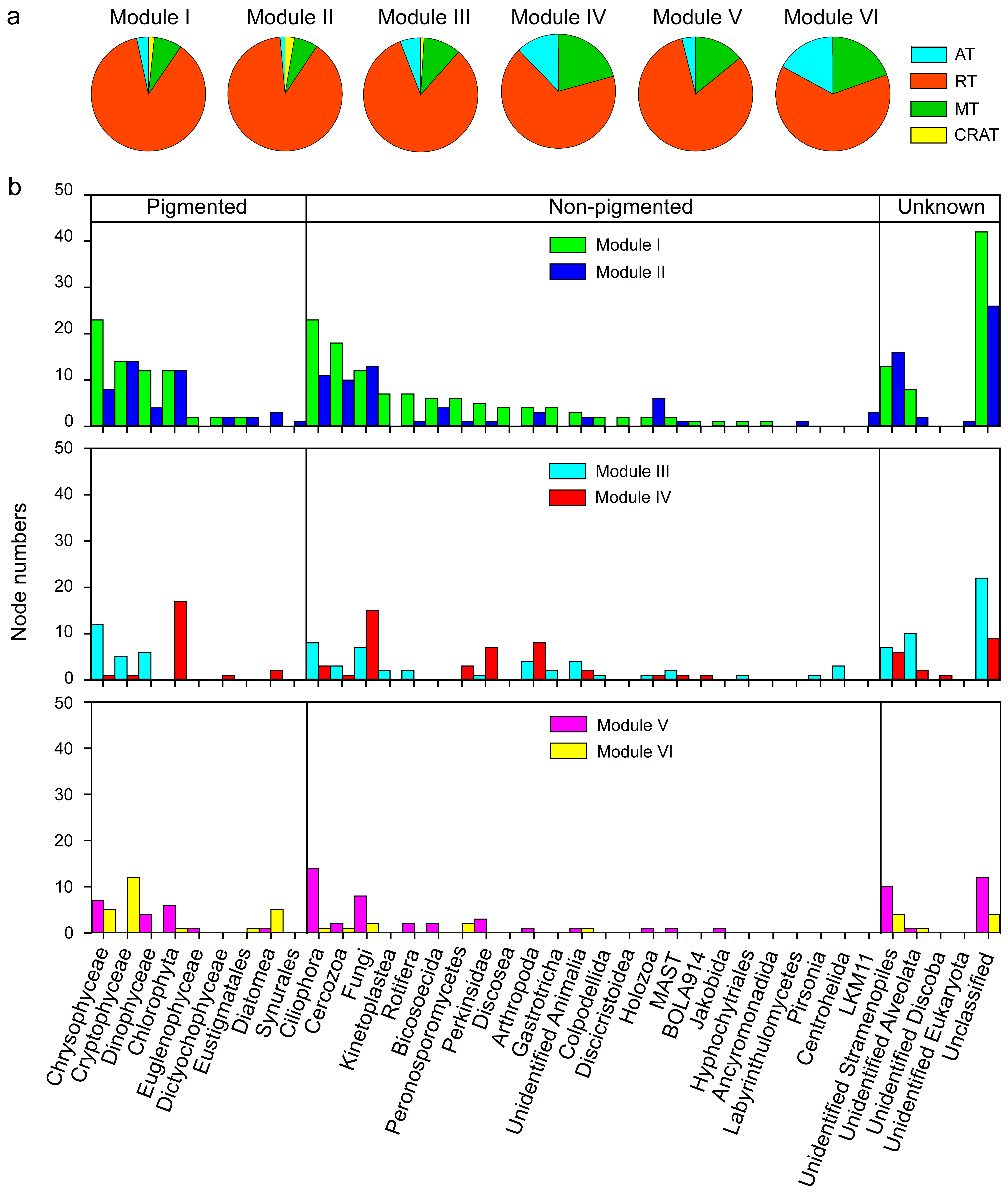


**Fig. S10** Components of eukaryotic plankton biodiversity in each module of the co-occurrence network. **a** Pie charts showing the richness proportion of different subcommunities. **b** Composition and number of plankton OTUs in each module of the co-occurrence network. AT abundant taxa, RT rare taxa, MT moderate taxa, CRAT conditionally rare and abundant taxa.


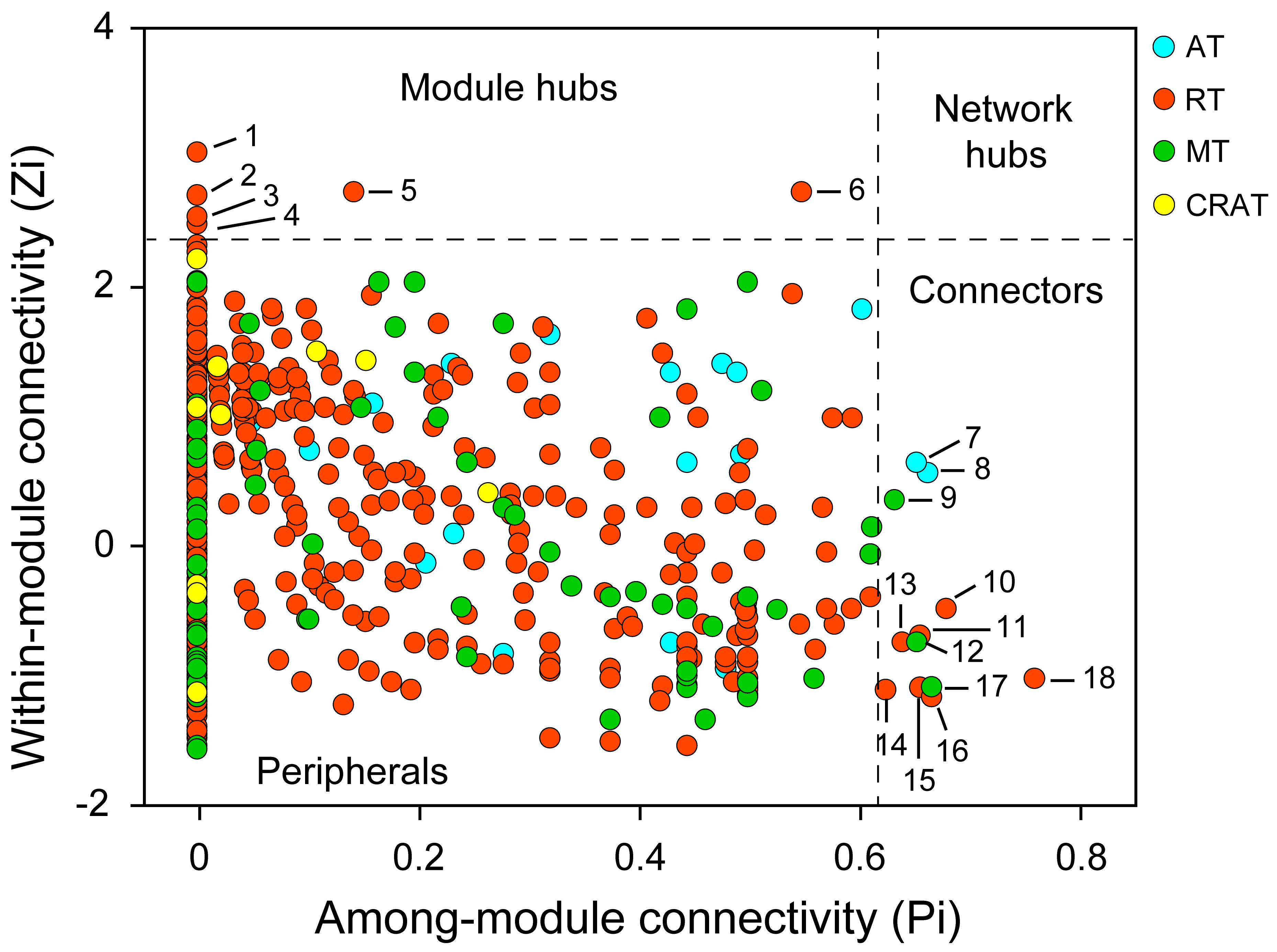


**Fig. S11** Zi-Pi plot showing the distribution of OTUs based on their topological roles. Each symbol represents an OTU. The topological role of each OTU was determined according to the scatter plot of within-module connectivity (Zi) and among-module connectivity (Pi). The module hubs and connectors are numbered and taxonomic rank is shown in Table S7. AT abundant taxa, RT rare taxa, MT moderate taxa, CRAT conditionally rare and abundant taxa.


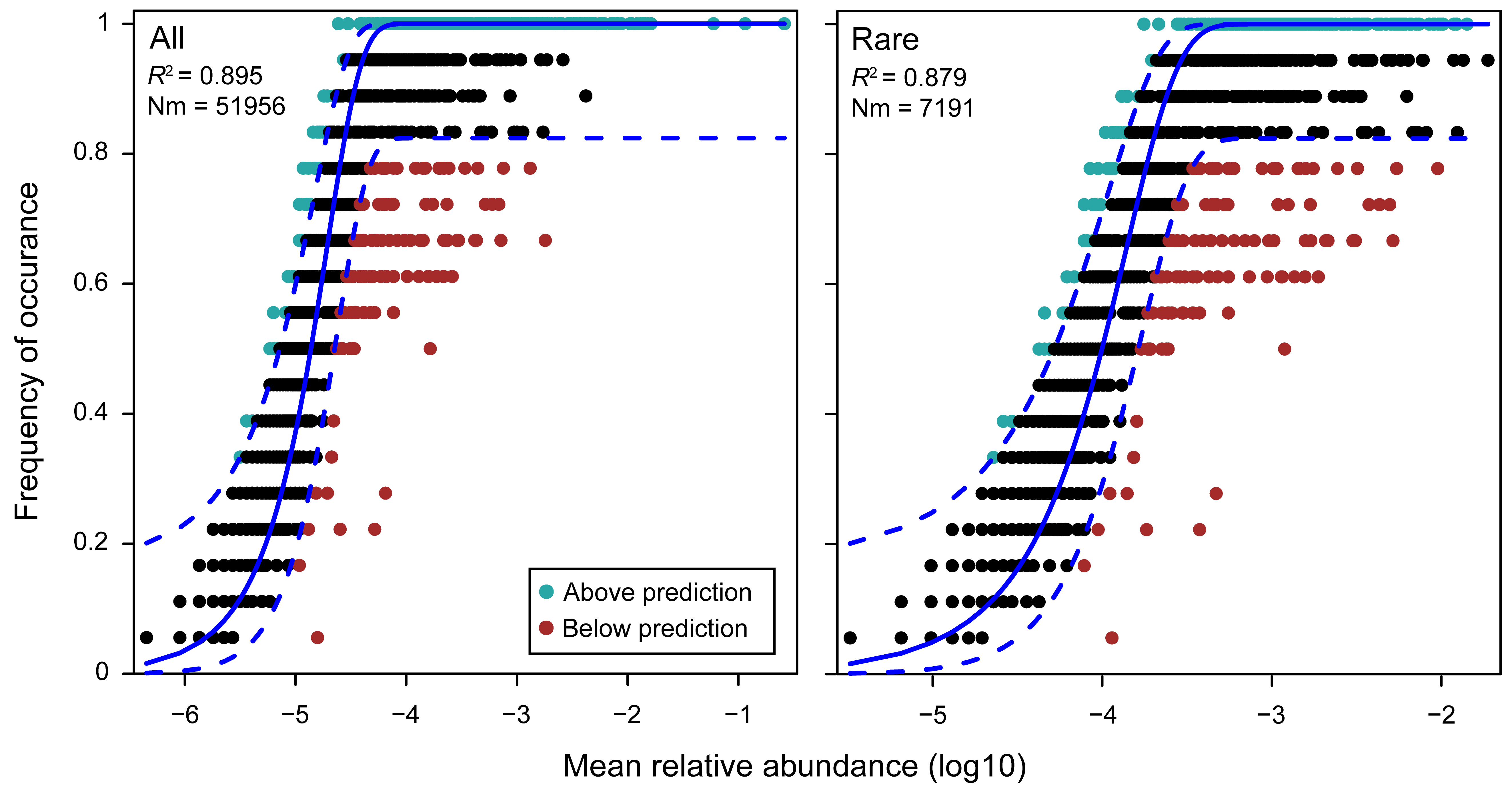


**Fig. S12** Fit of neutral model for all and rare eukaryotic plankton OTUs. The predicated occurrence frequency is shown as a solid blue line, and dashed blue lines represent 95% confidence intervals around the model prediction. OTUs that occur more or less frequently than predicted by the neutral model are shown in different colors. Nm indicates metacommunity size times immigration. *R*^2^ indicates the fit to the neutral model. All, whole eukaryotic plankton (*n* = 2305); Rare, rare eukaryotic plankton (*n* = 2167).


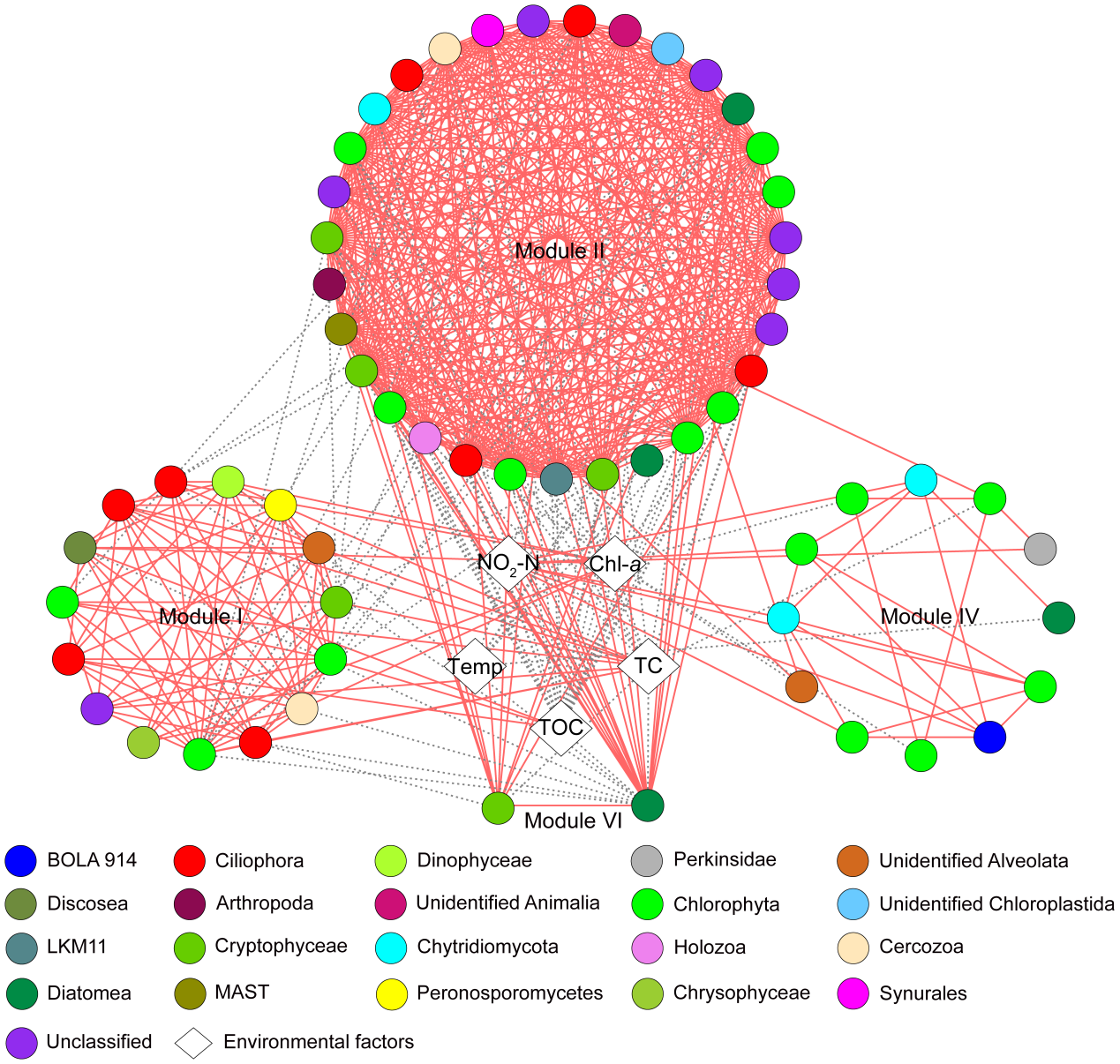


**Fig. S13** Species-species association network divided by module and species-environment association network. The OTUs from six major modules were included, and only correlations between species, and environmental factors and species that were significant (Spearman’s *r* > 0.8 or *r* < –0.8, and *P*-value < 0.01) were shown. Red solid and grey dotted lines indicate positive and negative correlations, respectively. Different taxonomic groups were represented with different colors. The species-species subnetworks were extracted from the entire co-occurrence network. Temp water temperature, TC total carbon, TOC total organic carbon, NO_2_-N nitrite nitrogen, Chl-*a* chlorophyll *a*.

**Table S1** Detailed description of abundant and rare plankton OTUs data sets at 97% similarity level

| Category | OTU number | Sequence number | Dissimilarity  contribution (%) |
| --- | --- | --- | --- |
| Always abundant taxa | 2 (0.09%) | 708,247 (31.97%) | 2.21 |
| Conditionally abundant taxa | 42 (1.82%) | 910,144 (41.08%) | 17.52 |
| Always rare taxa | 1524 (66.12%) | 19,503 (0.88%) | 20.90 |
| Conditionally rare taxa | 643 (27.90%) | 287,205 (12.96%) | 44.86 |
| Moderate taxa | 84 (3.64%) | 219,974 (9.93%) | 10.83 |
| Conditionally rare and abundant taxa | 10 (0.43%) | 70,547 (3.18%) | 3.68 |

Always abundant taxa (AAT) were defined as the OTUs with a relative abundance ≥1% in all samples

Conditionally abundant taxa (CAT) were defined as the OTUs with a relative abundance ≥1% in some samples but never rare (<0.01%) in any samples

Always rare taxa (ART) were defined as the OTUs with a relative abundance <0.01% in all samples

Conditionally rare taxa (CRT) were defined as the OTUs with a relative abundance <0.01% in some samples but never abundant (≥1%) in any samples

Moderate taxa (MT) were defined as the OTUs with a relative abundance between 0.01% and 1% in all samples

Conditionally rare and abundant taxa (CRAT) were defined as the OTUs with a relative abundance varying from rare (<0.01%) to abundant (≥1%)

**Table S2** General description of all, abundant and rare plankton OTUs data sets at 97% similarity level

| Category | OTU number | Sequence number | Chao1 | ACE |
| --- | --- | --- | --- | --- |
| All | 2305 | 2,215,620 | 2372 ± 15 | 2371 ± 24 |
| Abundant taxa | 44 (1.91%) | 1,618,391 (73.04%) |  |  |
| Rare taxa | 2167 (94.01%) | 306,708 (13.84%) |  |  |

All, whole eukaryotic plankton taxa

Abundant taxa (AT), including always abundant taxa (AAT) and conditionally abundant taxa (CAT)

Rare taxa (RT), including always rare taxa (ART) and conditionally rare taxa (CRT)

**Table S3** The numbers of all sequences, all OTUs, abundant OTUs and rare OTUs within each taxonomic group with and without chloroplasts

| Taxonomic groups | All sequences | All OTUs number | Abundant OTUs number | Rare OTUs number |
| --- | --- | --- | --- | --- |
| **Pigmented** |  |  |  |  |
| *Alveolata* |  |  |  |  |
| Dinophyceae | 41426 | 76 | 2 | 72 |
| *Archaeplastida* |  |  |  |  |
| Chlorophyta | 81394 | 117 | 3 | 102 |
| Glaucophyta | 3 | 1 |  | 1 |
| Phragmoplastophyta | 521 | 4 |  | 4 |
| Streptophyta | 61 | 5 |  | 5 |
| Others | 165 | 2 |  | 2 |
| *Excavata* |  |  |  |  |
| Euglenophyceae | 855 | 14 |  | 14 |
| *Stramenopiles* |  |  |  |  |
| Bolidomonas | 5 | 1 |  | 1 |
| Chrysophyceae | 122194 | 99 | 5 | 86 |
| Diatomea | 56380 | 36 | 2 | 32 |
| Dictyochophyceae | 801 | 9 |  | 9 |
| Eustigmatales | 1189 | 15 |  | 15 |
| Synurales | 96 | 3 |  | 3 |
| *Incertae sedis Eukaryota* |  |  |  |  |
| Cryptophyceae | 306410 | 94 | 7 | 80 |
| **Non-pigmented** |  |  |  |  |
| *Alveolata* |  |  |  |  |
| Apicomplexa | 144 | 10 |  | 10 |
| BOLA914 | 32874 | 7 | 2 | 5 |
| Ciliophora | 144488 | 205 | 6 | 190 |
| Colpodellida | 1272 | 3 |  | 2 |
| Oxyrrhis | 6 | 1 |  | 1 |
| Perkinsidae | 25524 | 21 | 1 | 18 |
| Unidentified Protalveolata | 61 | 2 |  | 2 |
| *Amoebozoa* |  |  |  |  |
| Discosea | 622 | 12 |  | 12 |
| Gracilipodida | 41 | 2 |  | 2 |
| LEMD255 | 4084 | 2 |  | 1 |
| LKM74 | 11 | 1 |  | 1 |
| Tubulinea | 46 | 3 |  | 3 |
| Others | 3 | 1 |  | 1 |
| *Excavata* |  |  |  |  |
| Diplonemea | 33 | 2 |  | 2 |
| Euglenida | 251 | 24 |  | 24 |
| Jakobida | 98 | 3 |  | 3 |
| Kinetoplastea | 8495 | 45 |  | 45 |
| Tetramitia | 53 | 4 |  | 4 |
| *Animalia* |  |  |  |  |
| Arthropoda | 988978 | 61 | 9 | 46 |
| Gastrotricha | 1359 | 10 |  | 10 |
| Platyhelminthes | 6816 | 3 | 1 | 2 |
| Rotifera | 59153 | 22 | 2 | 15 |
| Others | 15539 | 67 |  | 65 |
| *Opisthokonta* |  |  |  |  |
| Discicristoidea | 336 | 6 |  | 6 |
| Fungi | 31405 | 186 |  | 184 |
| Holozoa | 4740 | 19 |  | 19 |
| **Table S3** Continued |  |  |  |  |
| Taxonomic groups | All sequences | All OTUs number | Abundant OTUs number | Rare OTUs number |
| *Rhizaria* |  |  |  |  |
| Cercozoa | 55090 | 94 | 2 | 91 |
| Retaria | 4 | 1 |  | 1 |
| *Stramenopiles* |  |  |  |  |
| Bicosoecida | 11030 | 27 |  | 24 |
| Hyphochytriales | 2816 | 2 |  | 1 |
| Labyrinthulomycetes | 27 | 3 |  | 3 |
| MAST | 8217 | 12 |  | 9 |
| Peronosporomycetes | 10612 | 47 | 1 | 45 |
| Pirsonia | 352 | 1 |  | 1 |
| *Incertae sedis Eukaryota* |  |  |  |  |
| Ancyromonadida | 139 | 1 |  | 1 |
| Centrohelida | 2511 | 7 |  | 7 |
| LKM | 9955 | 9 |  | 8 |
| Rigifilida | 32 | 7 |  | 7 |
| **Unknown** |  |  |  |  |
| Unidentified Alveolata | 16469 | 76 |  | 73 |
| Unidentified Discoba | 240 | 30 |  | 30 |
| Unidentified Stramenopiles | 76917 | 128 | 1 | 117 |
| Unidentified Eukaryota | 3273 | 17 |  | 16 |
| Unclassified | 80004 | 645 |  | 634 |
| **Total** | 2215620 | 2305 | 44 | 2167 |

Others in each super-group include the rare lineages (OTUs < 3 and sequences < 100) and unidentified lineages

**Table S4** List of indicator species found during the bloom and post-bloom periods

| OTU ID | Association statistic | *P* | Category | Taxonomy |
| --- | --- | --- | --- | --- |
| **Bloom (*n* = 41)** | | | | |
| OTU_715 | 0.866 | 0.039* | ART | k_Alveolata |
| OTU_1185 | 0.826 | 0.011* | ART | k_Alveolata |
| OTU_596 | 0.909 | 0.001*** | ART | k_Alveolata;p_Ciliophora |
| OTU_842 | 0.791 | 0.020* | ART | k_Alveolata;p_Ciliophora;c_Heterotrichea;o_unidentified;f_unidentified;g_*Stentor*;s_uncultured ciliate |
| OTU_947 | 0.943 | 0.001*** | ART | k_Alveolata;p_Ciliophora;c_Litostomatea;o_unidentified;f_unidentified |
| OTU_455 | 0.891 | 0.040* | CRT | k_Alveolata;p_Ciliophora;c_Oligohymenophorea;o_Conthreep;f_unidentified;g_*Telotrochidium*;s_uncultured ciliate |
| OTU_1364 | 0.816 | 0.021* | ART | k_Alveolata;p_Ciliophora;c_Oligohymenophorea;o_Conthreep;f_unidentified;g_*Tetrahymena* |
| OTU_780 | 0.872 | 0.012* | ART | k_Alveolata;p_Ciliophora;c_Spirotrichea;o_unidentified;f_unidentified |
| OTU_903 | 0.882 | 0.003** | ART | k_Alveolata;p_Protalveolata;c_Colpodellida;o_unidentified;f_unidentified;g_*Colpodella*;s_uncultured *Colpodella* |
| OTU_746 | 0.861 | 0.018* | ART | k_Alveolata;p_unidentified;c_Dinophyceae |
| OTU_1166 | 0.775 | 0.043* | ART | k_Alveolata;p_unidentified;c_Dinophyceae |
| OTU_849 | 0.922 | 0.003** | ART | k_Amoebozoa;p_Tubulinea;c_Echinamoebida;o_unidentified;f_unidentified;g_*Echinamoeba*;s_uncultured eukaryote |
| OTU_1225 | 0.816 | 0.010** | ART | k_Animalia |
| OTU_1371 | 0.770 | 0.037* | ART | k_Animalia;p_Arthropoda;c_Maxillopoda;o_unidentified;f_unidentified;g_unidentified |
| OTU_1411 | 0.816 | 0.020* | ART | k_Chloroplastida;p_Chlorophyta;c_Chlorophyceae;o_unidentified;f_unidentified;g_*Carteria*;s_uncultured eukaryote |
| OTU_689 | 0.855 | 0.040* | CRT | k_Chloroplastida;p_Chlorophyta;c_Chlorophyceae;o_unidentified;f_unidentified;g_*Chlamydomonas*;s_*Chlamydomonas reinhardtii* |
| OTU_1553 | 0.816 | 0.018* | ART | k_Discoba |
| OTU_582 | 0.891 | 0.023* | CRT | k_Discoba;p_Euglenophyceae;c_unidentified;o_unidentified;f_Euglenaceae;g_*Euglena*;s_*Euglena agilis* |
| OTU_856 | 0.913 | 0.004** | ART | k_Discoba;p_Euglenozoa;c_Kinetoplastea;o_Metakinetoplastina;f_Neobodonida;g_*Rhynchobodo* |
| OTU_760 | 0.872 | 0.005** | ART | k_Eukaryota;p_unidentified;c_Cryptophyceae;o_Cryptomonadales;f_unidentified;g_*Cryptomonas* |
| OTU_636 | 0.869 | 0.026* | ART | k_Eukaryota;p_unidentified;c_Cryptophyceae;o_unidentified;f_unidentified;g_*Goniomonas*;s_*Goniomonas*_sp. SH-8 |
| OTU_874 | 0.830 | 0.046* | ART | k_Fungi;p_Ascomycota;c_Eurotiomycetes;o_Chaetothyriales;f_unidentified;g_unidentified |
| OTU_628 | 0.900 | 0.008** | CRT | k_Fungi;p_Chytridiomycota;c_Chytridiomycetes |
| OTU_2136 | 0.816 | 0.016* | ART | k_Holozoa;p_Choanomonada |
| OTU_588 | 0.913 | 0.008** | ART | k_Rhizaria;p_Cercozoa |
| OTU_528 | 0.898 | 0.016* | CRT | k_Rhizaria;p_Cercozoa |
| OTU_579 | 0.903 | 0.011* | CRT | k_Rhizaria;p_Cercozoa;c_Thecofilosea;o_unidentified;f_unidentified;g_*Pseudodifflugia*;s_*Pseudodifflugia* cf. *gracilis* |
| OTU_1296 | 0.775 | 0.030* | ART | k_Rhizaria;p_Cercozoa;c_unidentified;o_unidentified;f_Vampyrellidae |
| OTU_1010 | 0.775 | 0.029* | ART | k_Stramenopiles;p_Diatomea;c_Bacillariophyceae;o_unidentified;f_unidentified;g_*Pinnularia* |
| OTU_591 | 0.923 | 0.003** | CRT | k_Stramenopiles;p_Peronosporomycetes;c_unidentified;o_unidentified;f_unidentified |
| OTU_961 | 0.863 | 0.001*** | ART | k_Stramenopiles;p_Peronosporomycetes;c_unidentified;o_unidentified;f_unidentified |
| OTU_619 | 0.843 | 0.036* | ART | k_Stramenopiles;p_Peronosporomycetes;c_unidentified;o_unidentified;f_unidentified |
| OTU_1103 | 0.850 | 0.016* | ART | k_Stramenopiles;p_unidentified;c_P34.45;o_unidentified;f_unidentified;g_unidentified;s_Chrysophyceae sp. EK-2010a |
| OTU_943 | 0.913 | 0.002** | ART | Unclassified |
| OTU_602 | 0.909 | 0.009** | CRT | Unclassified |
| **Table S4 Continued** | | | | |
| OTU ID | Association statistic | *P* | Category | Taxonomy |
| OTU_981 | 0.877 | 0.006** | ART | Unclassified |
| OTU_622 | 0.854 | 0.017* | CRT | Unclassified |
| OTU_1015 | 0.845 | 0.007** | ART | Unclassified |
| OTU_1400 | 0.816 | 0.018* | ART | Unclassified |
| OTU_894 | 0.809 | 0.041* | ART | Unclassified |
| OTU_1313 | 0.770 | 0.029* | ART | Unclassified |
| **Post-bloom 1 (*n* = 3)** | | | | |
| OTU_1168 | 0.745 | 0.046* | ART | k_Fungi;p_Ascomycota;c_Dothideomycetes;o_Pleosporales;f_unidentified;g_unidentified;s_uncultured eukaryote |
| OTU_964 | 0.913 | 0.001*** | ART | k_Stramenopiles;p_Peronosporomycetes;c_unidentified;o_unidentified;f_unidentified |
| OTU_655 | 0.903 | 0.019* | CRT | k_Stramenopiles;p_unidentified;c_Dictyochophyceae;o_Pedinellales;f_unidentified;g_*Pseudopedinella* |
| **Post-bloom 2 (*n* = 87)** | | | | |
| OTU_642 | 0.927 | 0.001*** | ART | k_Alveolata |
| OTU_214 | 0.926 | 0.004** | CRT | k_Alveolata |
| OTU_450 | 0.910 | 0.008** | CRT | k_Alveolata |
| OTU_502 | 0.909 | 0.008** | CRT | k_Alveolata |
| OTU_259 | 0.901 | 0.047* | CRT | k_Alveolata |
| OTU_363 | 0.894 | 0.017* | CRT | k_Alveolata |
| OTU_816 | 0.735 | 0.020* | ART | k_Alveolata;p_Ciliophora |
| OTU_1149 | 0.870 | 0.015* | ART | k_Alveolata;p_Ciliophora;c_Litostomatea;o_unidentified;f_unidentified |
| OTU_135 | 0.916 | 0.026* | CRT | k_Alveolata;p_Ciliophora;c_Oligohymenophorea;o_Conthreep;f_unidentified |
| OTU_276 | 0.897 | 0.017* | CRT | k_Alveolata;p_Ciliophora;c_Prostomatea;o_Conthreep;f_unidentified;g_*Cryptocaryon* |
| OTU_419 | 0.933 | 0.003** | CRT | k_Alveolata;p_Ciliophora;c_Spirotrichea;o_unidentified;f_unidentified |
| OTU_572 | 0.888 | 0.006** | CRT | k_Alveolata;p_Ciliophora;c_Spirotrichea;o_unidentified;f_unidentified |
| OTU_824 | 0.778 | 0.035* | ART | k_Alveolata;p_Ciliophora;c_Spirotrichea;o_unidentified;f_unidentified;g_*Anteholosticha*;s_*Metaurostylopsis* sp. WWS-2010b |
| OTU_149 | 0.911 | 0.045* | CRT | k_Alveolata;p_Ciliophora;c_Spirotrichea;o_unidentified;f_unidentified;g_uncultured |
| OTU_2154 | 0.909 | 0.004** | CRT | k_Alveolata;p_Ciliophora;c_Spirotrichea;o_unidentified;f_unidentified;g_uncultured |
| OTU_1199 | 0.949 | 0.002** | ART | k_Alveolata;p_Protalveolata;c_unidentified;o_A31;f_Perkinsidae;g_unidentified;s_uncultured alveolate |
| OTU_609 | 0.921 | 0.001*** | CRT | k_Alveolata;p_Protalveolata;c_unidentified;o_unidentified;f_Perkinsidae;g_*Parvilucifera*;s_uncultured alveolate |
| OTU_594 | 0.903 | 0.010** | CRT | k_Alveolata;p_unidentified;c_Dinophyceae |
| OTU_969 | 0.760 | 0.009** | ART | k_Alveolata;p_unidentified;c_Dinophyceae;o_Gymnodiniphycidae |
| OUT_897 | 0.970 | 0.002** | ART | k_Animalia |
| OTU_536 | 0.872 | 0.008** | CRT | k_Animalia;p_Gastrotricha;c_unidentified;o_unidentified;f_Chaetonotidae;g_unidentified |
| OTU_664 | 0.859 | 0.020* | CRT | k_Animalia;p_unidentified;c_unidentified;o_unidentified;f_unidentified;g_unidentified |
| OTU_1424 | 0.924 | 0.010** | CRT | k_Centrohelida;p_unidentified;c_unidentified;o_unidentified;f_Heterophryidae |
| OTU_834 | 0.857 | 0.007** | ART | k_Chloroplastida;p_Chlorophyta |
| OTU_970 | 0.833 | 0.012* | ART | k_Chloroplastida;p_Chlorophyta |
| **Table S4 Continued** | | | | |
| OTU ID | Association statistic | *P* | Category | Taxonomy |
| OTU_877 | 0.816 | 0.018* | ART | k_Chloroplastida;p_Chlorophyta;c_Chlorophyceae;o_Elev-18S-713;f_unidentified;g_unidentified;s_uncultured Eimeriidae |
| OTU_1587 | 0.907 | 0.002** | ART | k_Chloroplastida;p_Chlorophyta;c_Chlorophyceae;o_unidentified;f_unidentified;g_*Chlamydomonas*;s_uncultured Chlorophyta |
| OTU_690 | 0.905 | 0.007** | ART | k_Chloroplastida;p_Phragmoplastophyta;c_unidentified;o_Zygnematales;f_unidentified;g_*Spirotaenia* |
| OTU_1991 | 0.935 | 0.001*** | ART | k_Eukaryota;p_P1-31;c_Cryptophyceae;o_unidentified;f_unidentified;g_unidentified;s_uncultured eukaryote |
| OTU_735 | 0.849 | 0.021* | ART | k_Eukaryota;p_unidentified;c_Cryptophyceae;o_Cryptomonadales;f_unidentified;g_*Cryptomonas*;s_*Cryptomonas curvata* |
| OTU_882 | 0.937 | 0.013* | CRT | k_Eukaryota;p_unidentified;c_Cryptophyceae;o_unidentified;f_Kathablepharidae;g_uncultured;s_uncultured Katablepharidaceae |
| OTU_554 | 0.924 | 0.002** | CRT | k_Fungi |
| OTU_640 | 0.886 | 0.011* | CRT | k_Fungi |
| OTU_733 | 0.826 | 0.033* | ART | k_Fungi;p_Ascomycota |
| OTU_835 | 0.842 | 0.005** | ART | k_Fungi;p_Chytridiomycota |
| OTU_1030 | 0.894 | 0.012* | CRT | k_Fungi;p_Chytridiomycota;c_Chytridiomycetes;o_Rhizophydiales;f_unidentified |
| OTU_2142 | 0.849 | 0.024* | CRT | k_Fungi;p_Chytridiomycota;c_Chytridiomycetes;o_Rhizophydiales;f_unidentified |
| OTU_1688 | 0.919 | 0.001*** | CRT | k_Holozoa;p_Choanomonada;c_Craspedida;o_unidentified;f_Codonosigidae;g_*Sphaeroeca*;s_*Sphaeroeca volvox* |
| OTU_236 | 0.889 | 0.024* | CRT | k_Holozoa;p_Choanomonada;c_unidentified;o_unidentified;f_unidentified;g_unidentified;s_uncultured Eimeriidae |
| OTU_741 | 0.910 | 0.001*** | ART | k_Rhizaria;p_Cercozoa |
| OTU_79 | 0.932 | 0.013* | CRAT | k_Rhizaria;p_Cercozoa;c_Thecofilosea;o_unidentified;f_unidentified;g_uncultured;s_uncultured eukaryote |
| OTU_999 | 0.913 | 0.005** | ART | k_Rhizaria;p_Cercozoa;c_unidentified;o_unidentified |
| OTU_180 | 0.894 | 0.017* | CRT | k_Stramenopiles |
| OTU_629 | 0.835 | 0.009** | CRT | k_Stramenopiles |
| OTU_1592 | 0.913 | 0.004** | ART | k_Stramenopiles;p_MAST-12;c_unidentified;o_unidentified;f_unidentified;g_unidentified;s_uncultured Eimeriidae |
| OTU_480 | 0.932 | 0.001*** | CRT | k_Stramenopiles;p_unidentified |
| OTU_121 | 0.923 | 0.012* | CRT | k_Stramenopiles;p_unidentified |
| OTU_1151 | 0.913 | 0.001*** | ART | k_Stramenopiles;p_unidentified |
| OTU_1035 | 0.842 | 0.049* | ART | k_Stramenopiles;p_unidentified |
| OTU_2162 | 0.827 | 0.021* | ART | k_Stramenopiles;p_unidentified |
| OTU_773 | 0.802 | 0.024* | ART | k_Stramenopiles;p_unidentified |
| OTU_253 | 0.904 | 0.008** | CRT | k_Stramenopiles;p_unidentified;c_Chrysophyceae |
| OTU_281 | 0.900 | 0.010** | CRT | k_Stramenopiles;p_unidentified;c_Chrysophyceae |
| OTU_796 | 0.859 | 0.004** | ART | k_Stramenopiles;p_unidentified;c_Chrysophyceae |
| OTU_978 | 0.816 | 0.011* | ART | k_Stramenopiles;p_unidentified;c_Chrysophyceae |
| OTU_800 | 0.805 | 0.049* | ART | k_Stramenopiles;p_unidentified;c_Chrysophyceae |
| OTU_526 | 0.922 | 0.001*** | CRT | k_Stramenopiles;p_unidentified;c_Chrysophyceae;o_Ochromonadales;f_unidentified;g_*Paraphysomonas* |
| OTU_711 | 0.850 | 0.004** | ART | k_Stramenopiles;p_unidentified;c_Chrysophyceae;o_Ochromonadales;f_unidentified;g_*Paraphysomonas*;s_uncultured chrysophyte |
| OTU_756 | 0.861 | 0.005** | ART | k_Stramenopiles;p_unidentified;c_Dictyochophyceae;o_Pedinellales;f_unidentified |
| OTU_1192 | 0.961 | 0.003** | CRT | k_Stramenopiles;p_unidentified;c_Dictyochophyceae;o_Pedinellales;f_unidentified;g_*Pseudopedinella*;s_uncultured dictyochophyte |
| OTU_1143 | 0.866 | 0.008** | ART | k_Stramenopiles;p_unidentified;c_unidentified;o_Bicosoecida;f_Siluaniidae |
| **Table S4 Continued** | | | | |
| OTU ID | Association  statistic | *P* | Category | Taxonomy |
| OTU_843 | 0.851 | 0.006** | ART | k_Stramenopiles;p_unidentified;c_unidentified;o_Labyrinthulomycetes |
| OTU_1050 | 0.816 | 0.010** | ART | k_Stramenopiles;p_unidentified;c_unidentified;o_Synurales;f_unidentified |
| OTU_261 | 0.948 | 0.011* | CRT | Unclassified |
| OTU_263 | 0.925 | 0.023* | CRT | Unclassified |
| OTU_410 | 0.918 | 0.009** | CRT | Unclassified |
| OTU_938 | 0.913 | 0.005** | ART | Unclassified |
| OTU_1002 | 0.913 | 0.004** | ART | Unclassified |
| OTU_1807 | 0.910 | 0.002** | ART | Unclassified |
| OTU_283 | 0.901 | 0.016* | CRT | Unclassified |
| OTU_2207 | 0.899 | 0.002** | CRT | Unclassified |
| OTU_373 | 0.899 | 0.030* | CRT | Unclassified |
| OTU_225 | 0.897 | 0.016* | CRT | Unclassified |
| OTU_597 | 0.893 | 0.005** | CRT | Unclassified |
| OTU_295 | 0.890 | 0.027* | CRT | Unclassified |
| OTU_657 | 0.888 | 0.045* | CRT | Unclassified |
| OTU_803 | 0.887 | 0.005** | ART | Unclassified |
| OTU_492 | 0.879 | 0.042* | CRT | Unclassified |
| OTU_493 | 0.871 | 0.007** | CRT | Unclassified |
| OTU_909 | 0.866 | 0.016* | ART | Unclassified |
| OTU_918 | 0.850 | 0.018* | ART | Unclassified |
| OTU_986 | 0.833 | 0.005** | ART | Unclassified |
| OTU_889 | 0.816 | 0.018* | ART | Unclassified |
| OTU_905 | 0.816 | 0.018* | ART | Unclassified |
| OTU_1211 | 0.816 | 0.009** | ART | Unclassified |
| OTU_2011 | 0.816 | 0.018* | ART | Unclassified |
| OTU_2109 | 0.816 | 0.017* | ART | Unclassified |

ART always rare taxa, CRT conditionally rare taxa, CRAT conditionally rare and abundant taxa

**P* < 0.05, ***P* < 0.01, ****P* < 0.001

**Table S5** Topological properties of the real co-occurrence networks of eukaryotic plankton communities and their associated random networks

|  | Real networks | | | | | | |  | Random networks | | | |
| --- | --- | --- | --- | --- | --- | --- | --- | --- | --- | --- | --- | --- |
|  | Nodes ^a^ | Edges ^b^ | Modularity ^c^ | Average clustering coefficient ^d^ | Network diameter ^e^ | Average path length ^f^ | Average degree ^g^ |  | Modularity (SD) ^c^ | Average clustering coefficient (SD) ^d^ | Average path length (SD) ^f^ | Small-word coefficient  (SD) ^h^ |
| Whole | 791 | 9628 | 0.618 | 0.473 | 12 | 4.020 | 24.344 |  | 0.137  (± 0.003) | 0.031  (± 0.001) | 2.427  (± 0.001) | 9.277  (± 0.189) |

Random networks were generated by rewiring all of the links with the same numbers of nodes and edges to the real networks

The number in the brackets indicates the standard deviation of topological properties of the 1000 Erdös-Rényi random networks

^a^ Number of OTUs with the correlation *r* > 0.8 or *r* < –0.8 and statistical significance (*P*-value < 0.01)

^b^ Number of strong and significant correlations between nodes

^c^ Modularity >0.4 suggests that the network has a modular structure. It indicates that there are nodes in the network that are more densely connected between each other than with the rest of the network and that their density is noticeably higher than the graph’s average [3].

^d^ How nodes are embedded in their neighborhood, and the degree to which nodes tend to cluster together

^e^ The maximum distance between all possible pairs of nodes

^f^ The average number of steps along the shortest paths for all possible pairs of network [nodes](https://en.wikipedia.org/wiki/Node_(networking))

^g^ Node connectivity showing how many connections (on average) each node has to the other nodes in the network

^h^ Small-word coefficient >1 indicates “small-world” properties, that is, high interconnectivity and high efficiency [4].

**Table S6** Lists of keystone species in co-occurrence network

| OTU ID | Betweenness  centrality | Degree | Modularity  classes | Category | Taxonomy |
| --- | --- | --- | --- | --- | --- |
| OTU_201 | 967.9 | 105 | II | CRT | k_Alveolata;p_unidentified;c_Dinophyceae |
| OTU_174 | 898.2 | 103 | II | CRT | k_Chloroplastida;p_Chlorophyta;c_Chlorophyceae;o_unidentified;f_unidentified |
| OTU_107 | 1304.6 | 103 | II | CRT | k_Chloroplastida;p_Chlorophyta;c_Chlorophyceae;o_unidentified;f_unidentified |
| OTU_112 | 1580.7 | 108 | II | CRT | k_Eukaryota;p_P1-31;c_Cryptophyceae;o_unidentified;f_unidentified;g_unidentified |
| OTU_64 | 714. 1 | 104 | II | CRT | k_Eukaryota;p_unidentified;c_Cryptophyceae;o_Cryptomonadales;f_unidentified;g_*Cryptomonas* |
| OTU_38 | 3695.9 | 103 | II | CAT | k_Rhizaria;p_Cercozoa |
| OTU_517 | 3153.0 | 106 | II | CRT | k_Rhizaria;p_Cercozoa;c_Imbricatea;o_Thaumatomonadida;f_unidentified;g_*Gyromitus* |
| OTU_375 | 905.2 | 101 | II | CRT | k_Stramenopiles |
| OTU_121 | 1000.8 | 102 | II | CRT | k_Stramenopiles;p_unidentified |
| OTU_49 | 1405.0 | 105 | II | CRAT | k_Stramenopiles;p_unidentified |
| OTU_1180 | 2627.4 | 104 | II | CRT | k_Stramenopiles;p_unidentified |
| OTU_284 | 694.0 | 102 | II | CRT | k_Stramenopiles;p_unidentified;c_Chrysophyceae |
| OTU_118 | 1873.8 | 105 | II | CRT | k_Stramenopiles;p_unidentified;c_Chrysophyceae;o_Chromulinales;f_unidentified |
| OTU_167 | 1306.1 | 104 | II | CRT | k_Stramenopiles;p_unidentified;c_Chrysophyceae;o_Ochromonadales;f_unidentified;g_*Paraphysomonas* |
| OTU_263 | 1852.5 | 105 | II | CRT | Unclassified |
| OTU_278 | 734.6 | 106 | II | CRT | Unclassified |
| OTU_349 | 611.3 | 104 | II | CRT | Unclassified |

Nodes with high degree (>100) and low betweenness centrality values (<5000) are recognized as keystone species in co-occurrence network [5].

CAT conditionally abundant taxa, CRT conditionally rare taxa, CRAT conditionally rare and abundant taxa

**Table S7** Lists of module hubs and connectors in co-occurrence network

| Number of nodes | OTU ID | Zi | Pi | Modularity  classes | Category | Taxonomy |
| --- | --- | --- | --- | --- | --- | --- |
| 1 | OTU_890 | 3.020 | 0.000 | Other | ART | Unclassified |
| 2 | OTU_183 | 2.689 | 0.000 | I | CRT | Unclassified |
| 3 | OTU_353 | 2.524 | 0.000 | I | CRT | k_Fungi |
| 4 | OTU_304 | 2.524 | 0.000 | I | CRT | Unclassified |
| 5 | OTU_1861 | 2.712 | 0.142 | IV | CRT | Unclassified |
| 6 | OTU_2082 | 2.712 | 0.548 | IV | CRT | k_Chloroplastida;p_Chlorophyta;c_Chlorophyceae;o_unidentified;f_unidentified;g_*Scenedesmus* |
| 7 | OTU_36 | 0.627 | 0.653 | IV | CAT | k_Chloroplastida;p_Chlorophyta;c_Mamiellophyceae;o_unidentified;f_unidentified;g_*Monomastix* |
| 8 | OTU_10 | 0.548 | 0.663 | VI | CAT | k_Stramenopiles;p_Diatomea;c_Mediophyceae;o_unidentified;f_unidentified;g_*Discostella* |
| 9 | OTU_992 | 0.338 | 0.633 | VI | MT | k_Stramenopiles;p_Diatomea;c_Mediophyceae;o_unidentified;f_unidentified;g_*Discostella* |
| 10 | OTU_862 | –0.502 | 0.680 | VI | CRT | k_Stramenopiles;p_Diatomea |
| 11 | OTU_1161 | –0.712 | 0.656 | VI | CRT | k_Alveolata |
| 12 | OTU_186 | –0.763 | 0.653 | IV | MT | k_Fungi;p_Chytridiomycota;c_Chytridiomycetes;o_Rhizophydiales;f_unidentified;g_uncultured |
| 13 | OTU_687 | –0.763 | 0.640 | IV | ART | k_Alveolata |
| 14 | OTU_925 | –1.131 | 0.625 | VI | CRT | k_Stramenopiles;p_unidentified;c_Chrysophyceae;o_Ochromonadales;f_unidentified;g_*Ochromonas* |
| 15 | OTU_1104 | –1.112 | 0.656 | V | CRT | Unclassified |
| 16 | OTU_744 | –1.186 | 0.667 | V | ART | Unclassified |
| 17 | OTU_1690 | –1.110 | 0.667 | IV | MT | k_Animalia;p_Arthropoda;c_Maxillopoda;o_unidentified;f_unidentified;g_unidentified |
| 18 | OTU_625 | –1.042 | 0.760 | I | CRT | k_Animalia;p_Gastrotricha;c_unidentified;o_unidentified;f_Chaetonotidae;g_unidentified |

Light gray area includes 6 module hubs, and white area includes 12 connectors in the co-occurrence network

Other indicates the OTU from a small module with nodes =17

Zi indicates within module connectivity, and Pi indicates among module connectivity. The nodes in a network could be divided into the following four subcategories according to their Zi and Pi values: (1) peripheral nodes (Zi < 2.5, Pi < 0.62), (2) connectors (Zi < 2.5, Pi ≥ 0.62), (3) module hubs (Zi ≥ 2.5, Pi < 0.62), and (4) network hubs (Zi ≥ 2.5, Pi ≥ 0.62) [6, 7].

CAT conditionally abundant taxa, ART always rare taxa, CRT conditionally rare taxa, MT moderate taxa

**References**

1. Xue YY, Yu Z, Chen HH, Yang JR, Liu M, Liu LM, et al. Cyanobacterial bloom significantly boosts hypolimnelic anammox bacterial abundance in a subtropical stratified reservoir. FEMS Microbiol Ecol. 2017; 93: fix118.

2. Yang JR, Lv H, Isabwea A, Liu LM, Yu XQ, Chen HH, et al. Disturbance-induced phytoplankton regime shifts and recovery of cyanobacteria dominance in two subtropical reservoirs. Water Res. 2017; 120: 52–63.

3. Newman MEJ. Modularity and community structure in networks. Proc Natl Acad Sci USA. 2006; 103: 8577–82.

4. Telesford QK, Joyce KE, Hayasaka S, Burdette JH, Laurienti PJ. The ubiquity of small-world networks. Brain Connectivity. 2011; 1: 367–75.

5. Ma B, Wang HZ, Dsouza M, Lou J, He Y, Dai ZM, et al. Geographic patterns of co-occurrence network topological features for soil microbiota at continental scale in eastern China. ISME J. 2016; 10: 1891–1901.

6. Guimerà R, Amaral LAN. Functional cartography of complex metabolic networks. Nature. 2005; 433: 895–900.

7. Deng Y, Jiang Y-H, Yang Y, He Z, Luo F, Zhou J. Molecular ecological network analyses. BMC Bioinformatics. 2012; 13:113.
